# The genome-scale sugar metabolic model from *Neurospora crassa* reveals lower gene redundancy than that of *Aspergillus niger*

**DOI:** 10.1016/j.crmicr.2026.100596

**Published:** 2026-04-15

**Authors:** Jiajia Li, Mao Peng, Bo Baas, Ruby E. Schnirman, Lori B. Huberman, Ronald P. de Vries

**Affiliations:** a Fungal Physiology, Westerdijk Fungal Biodiversity Institute, Uppsalalaan 8, 3584CT Utrecht, The Netherlands; b Plant Pathology and Plant-Microbe Biology Section, School of Integrative Plant Science, Cornell University, 307 Plant Science Building, Ithaca, NY 14853, USA; c Molecular Biology and Genetics, Cornell University, Ithaca, NY 14853, USA

**Keywords:** Sugar catabolic pathways, Plant biomass, Neurospora crassa, Filamentous fungi

## Abstract

The filamentous fungus *Neurospora crassa* has been a model organism for understanding many fundamental aspects of eukaryotic biology. Here, we constructed a genome-scale model of its sugar metabolism based on the sugar metabolic model of *Aspergillus niger* using an orthology-based approach. To further support the model, the role of the identified genes in specific pathways was validated by evaluating their expression in transcriptome data of *N. crassa* from previously published studies during growth on different carbon sources, and by growth phenotypes of deletion mutants for selected genes. This data was compared to another Sordariomycete model species (*Trichoderma reesei*) and the ascomycete *A. niger*.

The genome and orthology based sugar metabolic models revealed that *N. crassa* has high similarity to *T. reesei* at the genomic level, but a lower gene redundancy for individual pathway steps than *A. niger*. This was confirmed by significant growth reduction of strains in which a single gene for a pathway step was deleted, while previous studies in *A. niger* often required deletion of multiple genes to obtain significant growth reduction. Interestingly, there was higher similarity in the transcriptome profiles of orthologous genes from *T. reesei* and *A. niger*, while in *N. crassa*, different profiles were observed for genes of several pathways. This study provides a well-supported framework for metabolic studies in *N. crassa* and exemplifies the species-specific modification that occur in the organization of sugar metabolism in filamentous fungi.

## Highlights

• *Neurospora crassa* has lower sugar metabolic gene redundancy than *Aspergillus niger*.
• The mating type of *N. crassa* affects growth of sugar metabolic deletion strains.
• We present a strongly improved sugar metabolic model for *N. crassa*.

## Graphical abstract

**Figure:**
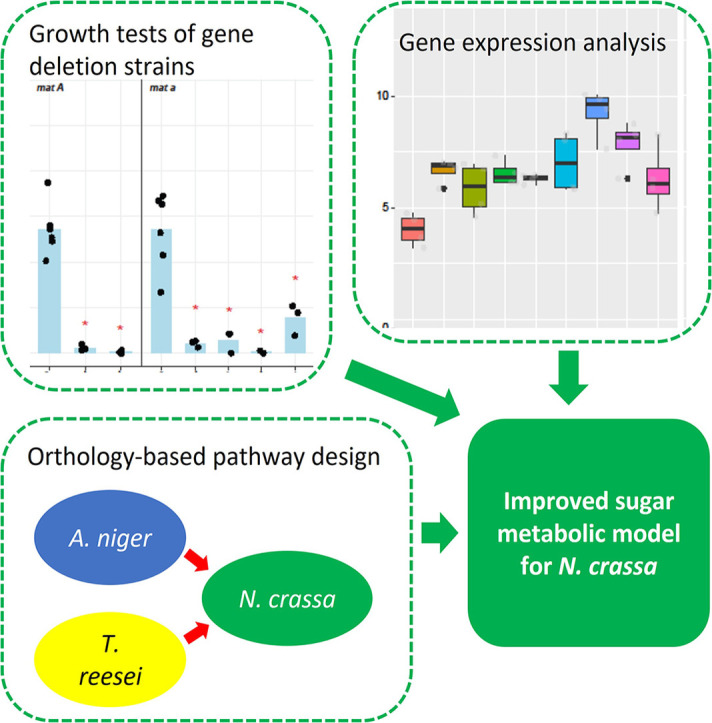

## 1 Introduction

Plant biomass represents an abundant and renewable resource on our planet. It plays a fundamental role as a primary raw material across various industries like chemical (e.g., solvents), food (sugar and sugar alcohols, etc.), and biofuel (ethanol, hydrogen, etc.) industries (Irmak, 2017; Kulikova et al., 2022). Fungi, in their natural environment, possess remarkable abilities to efficiently break down the complex polysaccharides present in plant biomass, releasing monosaccharides or small oligosaccharides. These released sugars are subsequently imported into the fungal cell and metabolized through diverse sugar metabolic pathways, enabling fungi to derive energy and sustain their growth (Wu et al., 2020). The ensemble of catabolic pathways that convert these sugars is known as primary carbon metabolism. A previous study generated a detailed metabolic model of the industrial workhorse *Aspergillus niger*, using the complete and manually annotated high quality genome of strain NRRL3 (Aguilar-Pontes et al., 2018). In this model, candidate genes for the different sugar metabolic pathways (using a combination of literature, GO terms and other annotations) were evaluated based on their expression on pathway-related carbon sources, and only genes with a positive correlation were maintained. Furthermore, the potential and limitations of an orthology-based transfer of the *A. niger* reference sugar metabolic model to other five fungi at different taxonomic distances from *A. niger* were investigated in a subsequent study (Li et al., 2022). The results revealed that the diversity of sugar metabolism correlates well with the taxonomic distance of the fungi. Transferring the sugar metabolic model from *A. niger* to *Trichoderma reesei* had limited success (Li et al., 2022).

The filamentous fungus *Neurospora crassa* is a widely studied species for genetic, cellular and biochemical research (Davis and Perkins, 2002; Galagan et al., 2003; Seiler and Plamann, 2003). Previous research in *N. crassa* has addressed the transcriptional and regulatory mechanisms governing carbon utilization, particularly during plant cell wall degradation (Benz et al., 2014; Coradetti et al., 2012; Sun et al., 2012; Thieme et al., 2017; Tian et al., 2009). In addition, the transcriptional response to major plant-derived monosaccharides have been addressed (Li et al., 2014; Wu et al., 2020), focusing particularly on the expression of genes encoding plant polysaccharide degrading enzymes (CAZymes). These studies revealed that *N. crassa* responds specifically to individual plant biomass components with some co-regulation of genes, likely reflecting the complex and heterogeneous nature of natural plant cell walls. In addition, while manually curated genome-scale metabolic models for *N. crassa* have been developed (Dreyfuss et al., 2013; Radford, 2004; Samal et al., 2017), they remain limited by significant knowledge gaps. Specifically, they include only glycolysis (previously described as the Embden–Meyerhof pathway), parts of the tricarboxylic acid and glyoxylate cycles, and the pentose phosphate pathway (PPP) (Dreyfuss et al., 2013; Radford, 2004). Moreover, the available information is somewhat fragmented and not fully integrated (Radford, 2004), and only some of the pathways for the degradation and utilization of plant cell wall polysaccharides were described (Samal et al., 2017).

To overcome these limitations and provide a comprehensive reference for future studies, in this project we applied an orthology-based approach to establish a detailed sugar metabolic model of *N. crassa*. It should be noted that this is a model of the organization of the pathways, not a metabolic flux model. This model incorporates all the current knowledge on fungal sugar metabolic pathways and therefore goes well beyond the metabolic models present in the KEGG website (https://www.genome.jp/kegg/). Comparing this model to the previously published models of *A. niger* and *T. reesei* enhances our understanding of the diversity of fungal sugar conversion, which will benefit further biochemical characterization and metabolic engineering of related fungi. It also raises awareness that even central biological processes may be organized differently between fungi, as demonstrated here for sugar metabolism, but which quite possibly is also true for other biological processes.

To validate the roles of sugar metabolic genes in the pathways of this model, we used RNA sequencing (RNAseq) data as well as phenotypes of relevant deletion mutants from the whole genome knockout library of *N. crassa* (Colot et al., 2006), going well beyond a mere genomic survey of possible pathways, by supporting the gene predictions by expression of the genes under relevant conditions and phenotypes of gene deletions on the related sugars. This revealed clear differences between *N. crassa, A. niger*, and *T. reesei*, highlighting the diversity of this basic biological process. Additionally, we expect that the sugar metabolic model of *N. crassa* will help to further explore its potential as a model organism and cell factory.

## 2 Methods

### 2.1 Strains, media and growth data

Three fungal species used in this study are *Aspergillus niger, Trichoderma reesei* and *Neurospora crassa*, and their details are denoted in Table 1. All *N. crassa* deletion mutants used in this study (Suppl. Table S1) were obtained from the previously constructed *N. crassa* full-genome deletion collection that was derived from the wild type reference strain using standard genetic techniques (Colot et al., 2006; McCluskey et al., 2010).

**Table 1:** List of species used in this study.

Species	References	Genome URL
*Aspergillus niger* NRRL3	(Aguilar-Pontes et al., 2018; Vesth et al., 2018)	https://mycocosm.jgi.doe.gov/Aspni_NRRL3_1/Aspni_NRRL3_1.home.html
*Trichoderma reesei* QM6a	(Li et al., 2017; Martinez et al., 2008)	https://mycocosm.jgi.doe.gov/Trire_Chr/Trire_Chr.home.html
*Neurospora crassa* OR74A v12.0	(Galagan et al., 2003)	https://mycocosm.jgi.doe.gov/Neucr2/Neucr2.home.html

For growth experiments, *N. crassa* cells were grown from freezer stocks on Vogel’s minimal medium (Vogel, 1956) + 2% sucrose + 1.5% agar (BD Difco 214010) slants for 2 days at 28 °C in the dark and 4 days at 28 °C in constant light before inoculation at 1 × 10^6^ conidia/mL into 3 mL of the indicated liquid medium in deep-well 24-well plates (Agilent Technologies 202061–100). The basic medium for all biomass growth experiments was a modified version of Vogel's minimal medium (Vogel, 1956) with ammonium as the nitrogen source (8.5 mM sodium citrate, 37 mM KH_2_PO_4_, 50 mM NH_4_Cl, 811 μM MgSO_4_, 680 μM CaCl_2_, 24 μM citric acid, 17 μM ZnSO_4_, 2.6 μM Fe(NH_4_)_2_(SO_4_)_2_, 1 μM CuSO_4_, 296 nM MnSO_4_, 809 nM H_3_BO_3_, 207 nM Na_2_MoO_4_, 20 nM biotin) and the indicated sugar added at 111 mM. Liquid cultures were incubated in constant light at 28 °C with constant shaking at 200 rpm for the following times: d-glucose (VWR 0188), 2 days; d-galactose (BeanTown Chemical 123650), 8 days; d-galacturonic acid (Thermo Fisher Scientific J66282.14), 13 days; l-rhamnose (AmBeed A827084), 4 days; l-arabinose (Chem-Impex 01654), 3 days; d-mannose (BeanTown Chemical 223055), 2 days; d-xylose (Thermo Fisher Scientific A10643.36), 2 days; and d-glucuronic acid (BeanTown Chemical 6556–12–3), 15 days. Incubation times for each carbon source were chosen such that the wild type strain had reached growth saturation shortly prior to harvesting biomass for each carbon source to allow for comparable growth of wild type cells across all carbon sources tested (Suppl. Table S3). Mycelial masses of deletion mutant cultures were harvested at the same timepoint as wild type cultures. Thus, differences in mycelial dry weight correspond to differences in growth speed between the wild type and mutant cultures. The pH of media containing d-glucuronic acid was adjusted to 5.8 and media containing d-galacturonic acid was pH 3.0. After the incubation period indicated above and in Suppl. Table S3 for each carbon source, the biomass of wild type and mutant strains were harvested by vacuum filtration onto Whatman Grade 1 filters (VWR 1001–055), washed, dried in a 70 °C drying oven for at least 24 h, and then weighed to determine the accumulated biomass. Filters were weighed before biomass was added and this value was subtracted from the final weight to determine mycelial dry weight. To control for any day-to-day differences in the lab environment that may have affected the final biomass weight (i.e., differences in temperature, relative humidity, etc.), filters without biomass were subjected to the same process in triplicate and the average difference in weight from the weighing prior to biomass harvest and on the date of weighing the biomass was subtracted from the accumulated biomass measurements. Relative accumulated biomass was determined by comparing the average biomass of the indicated strain grown on media containing glucose as the sole carbon source to the biomass of the biological replicates of the indicated strain grown on media containing the experimental carbon source inoculated on the same day. All strains grew comparably to the wild type strain on media containing glucose as the sole carbon source (Suppl. Fig. S1). Biological replicates were independently inoculated into liquid media on the same or different days. The number of biological replicates and the number of days across which the biological replicates were spread for each strain in each carbon source is indicated in Suppl. Table S3. Statistical significance was determined using a student’s *t*-test. Biomass data from all growth experiments is in Suppl. Table S4. The corresponding gene IDs of deletion mutations of *N. crassa* can be found in Suppl. Table S1.

For phenotypic analysis, a two-tailed distribution *t*-test was conducted to compare the reference strain to the metabolic deletion mutant strains grown on d-xylose, l-arabinose, l-rhamnose, d-galacturonic acid and d-galactose. Based on different biological replicates for each dataset, the *t*-test was evaluated with Microsoft Excel and parameters included the two-sample unequal variance. Further, the *t*-test score was expressed as P-value with the main assumption that if the value is < 0.05 the datasets are significantly different (marked in Fig. 4–7 with an asterisk *).

### 2.2 Identification of sugar metabolic genes in *N. crassa*

The protein and genome sequence data of *A. niger, T. reesei* and *N. crassa* were downloaded from the MycoCosm portal (Grigoriev et al., 2014) (Table 1). Gene names of *A. niger* metabolic genes were updated based on (de Vries & Li, under review). Additional functional annotations used to establish the sugar metabolic pathway in *N. crassa* were gathered from Gene Ontology (Ashburner et al., 2000), InterPro (Hunter et al., 2009; Paysan-Lafosse et al., 2023) and KEGG (Kanehisa and Goto, 2000) and were also obtained via MycoCosm portal. Transcriptome data of *N. crassa* was obtained from a previous study (Wu et al., 2020), except for the data in which *N. crassa* was exposed to glucose, which is from this study but performed identically to Wu et al. (2020).

Ortholog groups are identified using OrthoFinder version 2.5.4 (Emms and Kelly, 2019), default settings were used. OrthoFinder requires protein sequence fasta files from the desired organisms.

### 2.3 Metabolic model construction using pathway tools

The sugar metabolic pathway was built using Pathway Tools version 26.5 (Karp et al., 2021, 2002). PathoLogic of Pathway Tools is able to infer metabolic pathways and enzymes by analyzing the genome annotations with respect to reference databases of metabolic pathways like MetaCyc (Caspi et al., 2014). Firstly, we collected genome annotations, including KEGG annotation, GO annotation, KOG annotation, InterPro database annotation, genome functional information gff file, which are obtained from MycoCosm (Grigoriev et al., 2014), as well as the orthologs information. Then, we created a custom Perl pipeline to integrate all these annotations into a set of PathoLogic format (PF) files that can be recognized by the PathoLogic component of Pathway Tools software. Afterwards, we ran PathoLogic with standard settings (these take into account the genome complexity and data characteristics) to get the sugar metabolic model and manually modified it according to the ortholog mapping results in case that PathoLogic fails to assign a function based on annotation alone and removed the predicted genes with extremely low expression levels (maximum FPKM < 10 in the transcriptomes of all tested conditions).

### 2.4 Transcriptome analysis

The transcriptome data of *N. crassa* was obtained from a previous study (Wu et al., 2020), in which transcriptome profiling of *N. crassa* on multiple carbon sources was performed, except for the data in which *N. crassa* was exposed to glucose, which is from this study but performed identically to Wu et al. (2020). To generate the transcriptomic data in *N. crassa*, Wu et al. (Wu et al., 2020) inoculated 3 × 10^6^ 10 d old conidia into 3 ml of Vogel’s minimal medium (Vogel, 1956) + 2% sucrose in deep-well 24-well Whatman Uniplates. The bottoms of the wells for each plate were scratched with a sharp needle to enhance formation of a mycelial mat. After 16 h of incubation at 25 °C in constant light with shaking at 200 rpm, mycelia were washed three times in Vogel’s minimal medium (Vogel, 1956) without a carbon source and transferred to Vogel’s minimal medium (Vogel, 1956) with the indicated carbon source at 2 mM. After 4 h of incubation at 25 °C in constant light with shaking at 200 rpm, mycelia were harvested by filtering on Whatman #1 filter paper and flash frozen in liquid nitrogen prior to RNA extraction (Wu et al., 2020). The data of *A. niger* and *T. reesei* were described in the previous study (Li et al., 2022) and covered nine carbon sources (i.e., d-glucose, d-fructose, d-galactose, d-xylose, d-mannose, l-arabinose, d-galacturonic acid, d-glucuronic acid, l-rhamnose) for further analysis in this study. Transcript abundance was estimated in fragments per kilobase of transcript per million mapped reads (FPKMs) which were collected from the previously published expression data or was also analyzed using the Joint Genome Institute pipeline (Li et al., 2022; Wu et al., 2020). The normalized data from those studies was directly used to perform comparative analysis of the expression of the genes involved in sugar metabolism on these carbon sources. Differential expression analysis in *N. crassa* was performed on FPKMs with *limma*, version 3.65 (Ritchie et al., 2015), using data from biological triplicates, and the no carbon source was used as control. The threshold of log-transformed (base 2) fold change (log2FC) ≥ 1 or ≤ −1 and adjusted P-value < 0.01 was used to define significant differentially expressed genes (higher-/lower- expressed genes).

## 3 Results and discussion

**Fig. 1:**
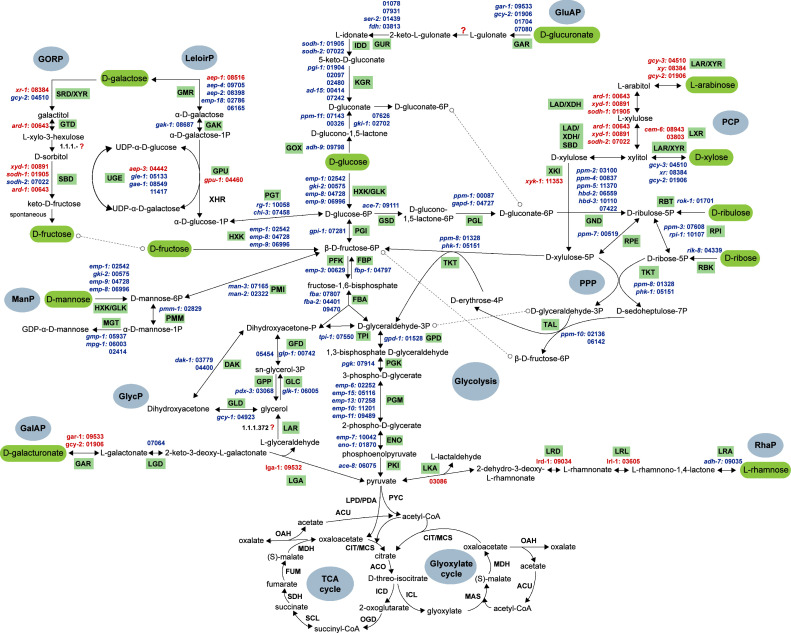
Sugar metabolic network in *N. crassa*. Overview of the sugar metabolic pathways with corresponding genes and reactions. Gene numbers are NCU numbers of *N. crassa*. Gene numbers highlighted in red are the genes selected for further experimental verification. Detailed phenotypic analysis can be found in Fig. 4, Fig. 5, Fig. 6, Fig. 7. Enzyme codes highlighted in green indicate the enzymes involved in each reaction and are fully described in Suppl. Table S2. Abbreviations in this figure: GORP: the d-galactose oxidoreductive pathway; LeloirP: Leloir pathway; ManP: d-mannose pathway; GalAP: d-galacturonic acid pathway; GlycP: Glycerol pathway; GluAP: d-glucuronic acid pathway; PCP: pentose catabolic pathway; PPP: Pentose phosphate pathway; RhaP: l-rhamnose pathway; TCA cycle: the tricarboxylic acid cycle.

**Fig. 2:**
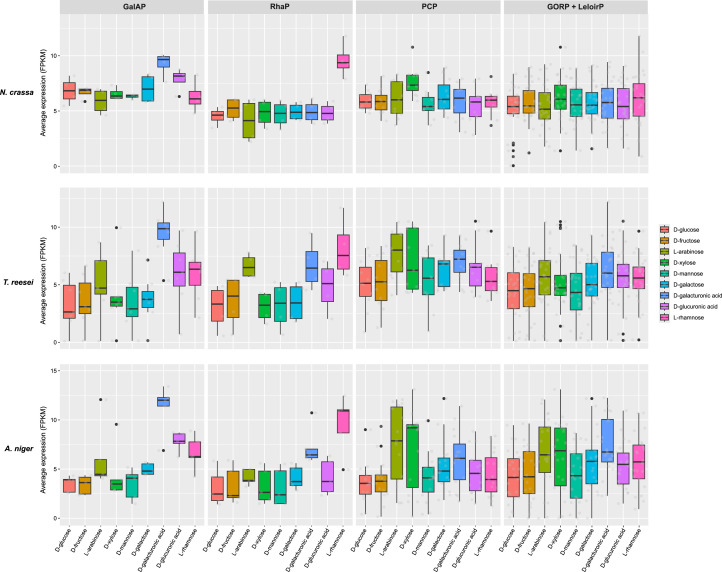
Change in transcription level of the genes assigned to four sugar metabolic pathways under nine carbon sources in *Aspergillus niger, Neurospora crassa* and *Trichoderma reesei*. Boxplots showing the change of expression of genes (FPKM (Fragments Per Kilobase of transcript per Million mapped reads) values) involved in different metabolic pathways depending on the carbon source used. The y-axis represents the average expression of genes involved in the corresponding pathway, and the x-axis depicts different monosaccharide conditions, and each grey small circle within each boxplot indicates an individual gene related to each specific sugar metabolic pathway.

**Fig. 3:**
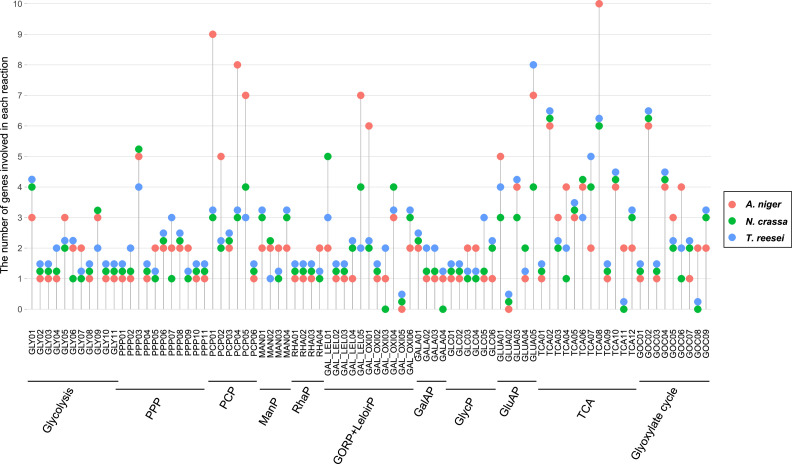
Distribution of gene content of each reaction from each sugar metabolic pathway across three fungal species. X-axis labels refer to the reaction designations, which can be found in Suppl. Table S2. The abbreviations of the names of pathways are consistent with the described name in Fig. 1. Different colored circles indicate different fungal species.

**Fig. 4:**
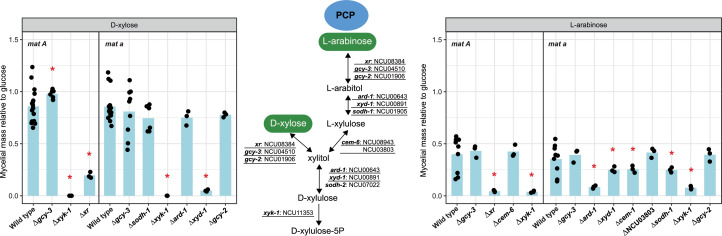
Pentose catabolic pathway (PCP) and phenotypic analysis of PCP-related genes during growth on d-xylose and l-arabinose in the wild type strains of *N. crassa* and deletion mutants. Statistically significant differences from the wild type strain (based on T-test, P < 0.05) are indicated by an asterisk. Underlined genes were chosen for phenotypic analysis.

**Fig. 5:**
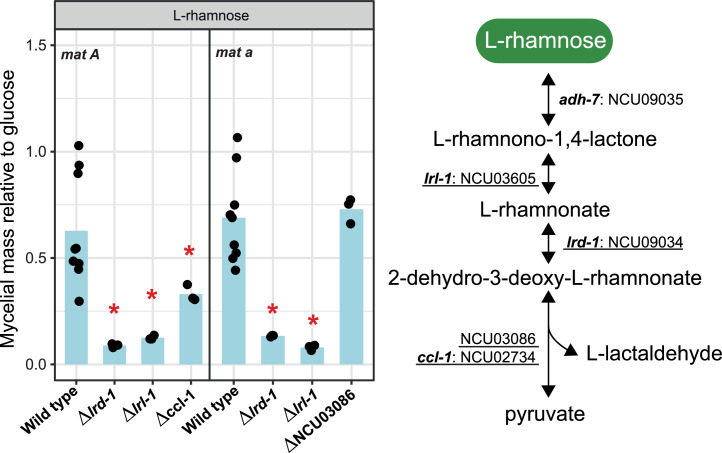
l-Rhamnose pathway (RhaP) and phenotypic analysis of RhaP-related genes during growth on l-rhamnose in the wild type strains of *N. crassa* and deletion mutants. Statistically significant differences from the wild type strain (based on T-test, P < 0.05) are indicated by an asterisk. Underlined genes were chosen for phenotypic analysis.

**Fig. 6:**
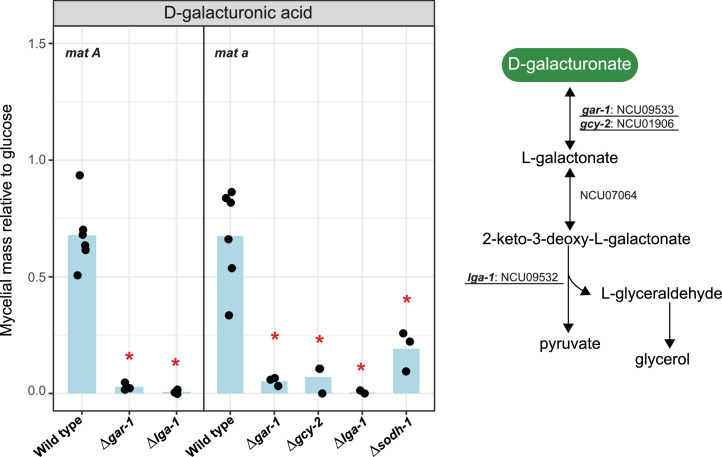
d-Galacturonic acid pathway (GalAP) and phenotypic analysis of GalAP-related genes during growth on d-galacturonic acid in the wild type strains of *N. crassa* and deletion mutants. Statistically significant differences from the wild type strain (based on T-test, P < 0.05) are indicated by an asterisk. Underlined genes were chosen for phenotypic analysis.

**Fig. 7:**
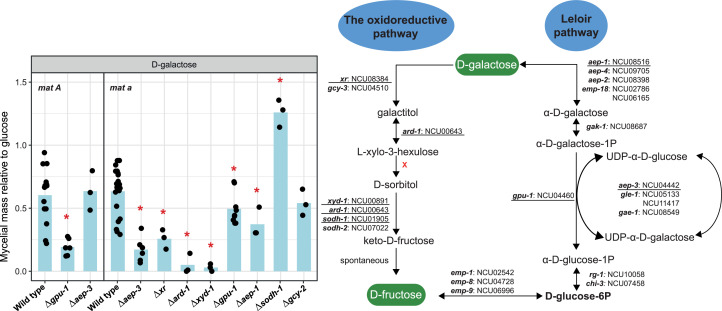
d-Galactose metabolism and phenotypic analysis of d-galactose metabolism-related genes during growth on d-galactose in the wild type strains of *N. crassa* and deletion mutants. Statistically significant differences from the wild type strain (based on T-test, P < 0.05) are indicated by an asterisk. Underlined genes were chosen for phenotypic analysis.

Orthologs of the *A. niger* sugar metabolic genes in *N. crassa* were identified using OrthoFinder (Emms and Kelly, 2019). Sugar metabolic pathways included in this study are: glycolysis, tricarboxylic acid (TCA) and glyoxylate cycles, pentose phosphate pathway (PPP), pentose catabolic pathway (PCP), l-rhamnose pathway (RhaP), d-galacturonic acid pathway (GaAP), d-galactose metabolism (Leloir pathway and d-galactose oxidoreductive pathway (GORP)), glycerol pathway (GlycP), d-mannose pathway (ManP), and d-glucuronic acid pathway (GluAP). An overview of the predicted sugar metabolic pathways in *N. crassa* is presented in Fig. 1 (details in Suppl. Table S2), and the expression of the genes per pathway (Fig. 2) and the number of genes per reaction step (Fig. 3) were analyzed. In addition, we evaluated single deletion mutants of selected sugar metabolic genes from PCP, RhaP, d-galactose metabolism, GalAP and ManP to verify their potential roles in sugar metabolism by measuring the mycelial mass relative to glucose (Fig. 4, Fig. 5, Fig. 6, Fig. 7). While we have not complemented these mutants again to demonstrate restoration of the while type phenotype, the good correlation between gene expression and deletion strain phenotype provides additional support for the roles of these genes in their respective pathways.

We present a new version of the sugar metabolic model, as previous published models (see Introduction) were mainly based on the KEGG database (Kanehisa et al., 2025), which has not incorporated the latest literature evidence. For instance, there is no specific pathway assigned to d-galacturonic acid metabolism in the KEGG database, and the galactose metabolic pathway (KEGG pathway: map00052) was not explicitly delineated into different sub-pathways. Some crucial reactions of PCP and PPP pathways (KEGG pathway: map00030 and map00040, respectively) were not well-annotated in the KEGG database. We limited this study to the sugar metabolic pathway rather than presenting a full genome-scale metabolic model, that integrates sugar pathways with lipid/nucleotide/amino acid metabolism, because there is not sufficient experimental data for most of the other pathways to achieve the same level of validation as we present here for sugar metabolism.

Overall, orthologs for most genes identified in *A. niger* and *T. reesei* (Li et al., 2022) were found in *N. crassa*. Some of these genes already had gene names that differ from those used in *A. niger* and *T. reesei*, and were therefore maintained in this study. A comparison of the gene names for the ortholog genes can be found in Suppl. Table S2. It may be advantageous to rename some of these genes in the future to increase uniformity and through that facilitate comparative studies, especially if they are performed using AI-based approaches (de Vries and Li, 2026).

### 3.1 Pentose catabolic pathway (PCP)

L-Arabinose and d-xylose are abundant pentoses found in plant cell wall components such as (arabino)xylan, xyloglucan, and pectin (Seiboth and Metz, 2011). In filamentous fungi, these pentoses are typically metabolized through the PCP (Witteveen et al., 1989) (Fig. 4).

Genes encoding or predicted to encode all key PCP enzymes were identified in *N. crassa. N. crassa* possessed a smaller PCP-related gene set than *A. niger*, but similar numbers as *T. reesei* (Fig. 3)*.* In *N. crassa*, based on homology with *A. niger* and *T. reesei*, we predicted NCU08384 and NCU04510 may also play a role in the reduction of l-arabinose and d-xylose. This prediction confirmed previous biochemical studies as NCU08384 was already shown to encode d-xylose reductase (named *xr*) (Woodyer et al., 2005; Zhao et al., 1998). NCU04510 is the ortholog of both LarA and XyrB from *A. niger* (Chroumpi et al., 2021; Mojzita et al., 2010) and was previously named *gcy-3* in *N. crassa* (Lamb et al., 2012). Previous studies (Bae et al., 2010; Sullivan and Zhao, 2007) identified and biochemically characterized NCU00643 (*ard-1*), which encodes an l-arabitol 4-dehydrogenase. NCU00891 (*xyd-1*) also exhibited homology to genes involved in the conversion of l-arabitol/xylitol to l-xylulose/D-xylulose. Similar to *T. reesei*, we did not identify gene copies encoding SBD in the *N. crassa* genome (Suppl. Table S2). However, we observed that growth of the deletion of NCU01905 (Δ*sodh-1*) was moderately reduced on l-arabinose, but not affected on d-xylose, suggesting that it is not involved in conversion of xylitol, but only in conversion of l-arabitol (Fig. 4). At the transcriptome level, the genes involved or predicted to be involved in the PCP displayed induction by l-arabinose and d-xylose in *A. niger* and *T. reesei*, while the PCP-related genes were predominantly induced by d-xylose in *N. crassa* (Fig. 2).

We hypothesized that genes without redundant function in the PCP would result in reduced growth on pentose sugars. To test this, we evaluated the growth of *N. crassa* strains lacking genes either known or predicted to play a role in the PCP. As expected, deletion of *xr, ard-1*, and NCU11353 (Δ*xyk-1*) resulted in significant reduced growth of *N. crassa* on l-arabinose (Fig. 4). However, deletion of NCU08943 (Δ*cem-6*), NCU03803 and *xyd-1* was not affected on growth on l-arabinose, which is similar to the case in *A. niger* where growth of Δ*lxrA* and Δ*xdhA* alone did not affect growth on l-arabinose (Chroumpi et al., 2021). It will be the role of future studies to investigate the phenotype of the Δ*cem-6* ΔNCU03803 double mutant to determine whether these genes play redundant roles. On d-xylose, the growth of Δ*xyk-1*, Δ*xr*, and Δ*xyd-1* deletion mutants was severely reduced (Fig. 4). Notably, deletion of *xyd-1* almost abolished growth on d-xylose in *N. crassa*, while growth of the *xdhA* deficient mutant (Δ*xdhA*) of *A. niger* was only slightly affected on d-xylose (Chroumpi et al., 2021). Since in *T. reesei* LAD1 appears to partially compensate for XDH1 activity (Seiboth et al., 2003), construction of a double deletion mutant Δ*ard-1*Δ*xyd-1* in *N. crassa* would reveal whether this is also the case in this fungus. Overall, according to the phenotype of the l-arabinose/D-xylose reductase mutants (Fig. 4), *xr* appears to be a critical enzyme for conversion of both l-arabinose and d-xylose into their respective polyols, which confirms a previous study in *N. crassa* (Li et al., 2014) and is similar to *T. reesei* (Akel et al., 2009). Compared to *A. niger, N. crassa* utilizes a partially different set of genes for the PCP, with *xr, ard-1, xyd-1*, and *xyk-1* serving as the dominant genes for the different pathway steps, whereas *A. niger* employs multiple enzymes with similar influence for nearly all steps of the pathway (Chroumpi et al., 2021). While it cannot be excluded that also in *N. crassa* additional not yet identified enzymes may contribute, overall, there seems to be a more prominent role for a single enzyme in each step, unlike the situation in *A. niger*.

### 3.2 l-Rhamnose pathway (RhaP)

L-Rhamnose is a hexose sugar abundantly present in both rhamnogalacturonan I (RG-I) and rhamnogalacturonan II (RG-II) (Chroumpi et al., 2020). After l-rhamnose is released, it is taken up into the fungal cell and converted into pyruvate and l-lactaldehyde in four enzymatic steps (Fig. 5), which are sequentially catalyzed by l-rhamnose-1-dehydrogenase (LRA), l-rhamnono-γ-lactonase (LRL), l-rhamnonate dehydratase (LRD) and l-2-keto-3-deoxyrhamnonate aldolase (LKA) (Chroumpi et al., 2020; Khosravi et al., 2017; Watanabe et al., 2008).

In *N. crassa*, we identified the orthologs of *A. niger lraA* (NCU09035, *adh-7*), *lrlA* (NCU03605, which we named *lrl-1* for l-rhamnono-γ-lactonase 1), *lrdA* (NCU09034, which we named *lrd-1* for l-rhamnonate dehydratase 1), and *lkaA* (NCU03086) (Fig. 5). Comparison of the expression levels of these four candidate genes on l-rhamnose and no carbon source showed that they were specifically induced on l-rhamnose (Thieme et al., 2017; Wu et al., 2020) (Suppl. Table S3). Furthermore, all four l-rhamnose pathway genes are regulated by the main pectinolytic regulator PDR-1 (the ortholog of *A. niger* RhaR (Gruben et al., 2014)) and were previously predicted to play a role in rhamnose catabolism (Thieme et al., 2017). In addition, *lrd-1* and *adh-7* are also regulated by the pectinolytic regulator PDR-2 (Wu et al., 2020), which is the ortholog of *A. niger* GaaR (Alazi et al., 2016). The RhaP is highly conserved in *A. niger, T. reesei* and *N. crassa*, with a single gene copy for each reaction at the genomic level (Fig. 3) and l-rhamnose–induced expression pattern of the corresponding genes at the transcriptomic level (Fig. 2).

Deletion of *lrd-1* and *lrl-1* both resulted in significantly reduced growth on l-rhamnose as sole carbon source, providing additional evidence that these two genes are involved in the l-rhamnose catabolic pathway (Fig. 5). Unfortunately, the *adh-7* deletion mutant is not in the *N. crassa* deletion collection, so its growth could not be tested (Colot et al., 2006). However, deletion of NCU03086 did not affect growth on l-rhamnose, suggesting either that this gene does not encode LKA in *N. crassa* or that there is more than one gene encoding LKA in *N. crassa*. In addition, we tested the growth of the NCU02734 deletion mutant because NCU02734 (*ccl-1*) and NCU03086 shared the HpcH/HpaI aldolase/citrate lyase domain (IPR005000, PF03328) according to InterPro (Hunter et al., 2009; Paysan-Lafosse et al., 2023) and PFAM (Mistry et al., 2021; Sonnhammer et al., 1997) databases. We observed a slight growth defect of the *ccl-1* deletion mutant on l-rhamnose (Fig. 5), suggesting that multiple genes may encode proteins with LKA activity or that the primary LKA-encoding gene(s) remain to be identified. Similarly, only the deletion of *lrlA* and *lrdA* completely abolished growth on l-rhamnose in *A. niger* (Chroumpi et al., 2020).

### 3.3 **D**-Galacturonic acid pathway (GalAP)

D-Galacturonic acid, a key component of pectin, is a naturally abundant carbon source for microorganisms living on decaying plant material (Kuorelahti et al., 2006; Richard and Hilditch, 2009). In fungi, the GalAP involves d-galacturonic acid reductase (GAR), l-galactonate dehydratase (LGD), and 2-keto-3-deoxy-l-galactonate aldolase (LGA) (Fig. 6) (Alazi et al., 2017; Mojzita et al., 2010). In *N. crassa*, the genes converting d-galacturonate to pyruvate and d-glyceraldehyde-3-phosphate were present and highly expressed on d-galacturonic acid (Suppl. Table S2 and Table S3). We predicted that two genes encode GAR, NCU09533, which we named *gar-1*, and *gcy-2* (NCU01906). Our analysis suggested that single genes encode for LGD (NCU07064) and LGA (NCU09532, which we named *lga-1*). Previous studies have predicted roles for *gar-1, gcy-2*, NCU07064, and *lga-1* in d-galacturonic acid utilization (Protzko et al., 2019; Wu et al., 2020). Unfortunately, we could not identify the ortholog of l-glyceraldehyde reductase encoding gene in *N. crassa*. Although a gene (NCU04923, *gcy-1*) was predicted to be the homolog of TrA2041C (GLD1) from *T. reesei* (Liepins et al., 2006) according to the OrthoFinder analysis and Blast, we do not have robust evidence to suggest its role in the GalAP. Compared to *T. reesei*, our homology search suggested that *N. crassa* had a reduced gene set involved in d-galacturonic acid pathway, and the *N. crassa* gene set appeared closer to the genes present in the *A. niger* genome (Fig. 3). Transcriptome analysis revealed that genes involved in d-galacturonic acid of all three species showed a consistent pattern of strong d-galacturonic acid induction (Fig. 2). Additionally, all four genes (*gar-1, gcy-2*, NCU07064, and *lga-1*) are regulated by the transcription factors PDR-1 and PDR-2 (Wu et al., 2020).

Phenotypic analysis revealed that Δ*gar-1*, Δ*gcy-2*, and Δ*lga-1* had significantly reduced growth on d-galacturonic acid compared to wild type strains (Fig. 6). In addition to the above genes, slightly reduced growth was also observed in the NCU01905 (*sodh-1*) deletion strain on d-galacturonic acid. This gene is an ortholog of NRRL3_00319 (*iddA*) from *A. niger*, and shared the same Pfam domain (PF00107) with other polyol dehydrogenases (LadA, LadB, XdhA, SdhA) of *A. niger*. A previous study in *A. niger* suggested that IddA was related to the PCP and the d-galactose oxidoreductive pathway with a more diverse metabolic role (Müller, 2024). The phenotypic analysis confirmed this result, indicating the similarity between *N. crassa* and *A. niger*. The *N. crassa* deletion collection (Colot et al., 2006) does not contain the homokaryotic deletion strain for NCU07064, but has a heterokaryotic strain with this deletion, suggesting that this gene may be essential for growth of *N. crassa*. This suggests that it has a more central role in *N. crassa* physiology and is not only involved in the d-galacturonic acid pathway. In contrast, two *A. niger* genes (*garA* and *garB*) were identified to be involved in the first step of the GalAP (Suppl. Table S2), but only the Δ*gar1* mutant showed a clear growth defect on d-galacturonic acid (Alazi et al., 2017).

### 3.4 **D**-Galactose metabolism

D-Galactose metabolism in filamentous fungi primarily proceeds via two routes: the Leloir pathway, and d-galactose oxidoreductive pathway (GORP). The Leloir pathway converts d-galactose into glucose-1-phosphate through a series of enzymatic steps involving galactose mutarotase (GMR), galactokinase (GAK), d-galactose-1-phosphate uridylyl transferase (GPU), UDP-galactose 4-epimerase (UGE), and phosphoglucomutase (PGL), thereby channeling it into central carbon metabolism. The GORP oxidizes d-galactose to d-galactonate, followed by further enzymatic steps leading to intermediates such as glyceraldehyde and pyruvate, which feed into glycolysis and the TCA cycle (Fig. 7).

In *N. crassa*, at least one gene encoding each of the enzymes of the Leloir pathway is present, and all genes required for the GORP are present except for a gene encoding l-xylo-3-hexulose reductase (XHR) (Fig. 7), which is different from *A. niger* and *T. reesei* (Fig. 3). Several of these genes have previously been characterized, including *xr* and *ard-1* discussed above and *rg-1* (NCU10058), which contributes to phosphoglucomutase activity in *N. crassa* (Brody and Tatum, 1967). As we observed, the distribution of gene content involved in d-galactose metabolism among the three species were variable. Furthermore, transcriptome analysis in *N. crassa* revealed that almost all predicted genes involved in this pathway were highly expressed under d-galactose growth conditions, as well as most other carbon sources (Fig. 2). Several of these genes (such as *ard-1* and NCU11417) are also regulated by *ara-1* (NCU05414), a transcription factor required for d-galactose and l-arabinose utilization in *N. crassa* (Wu et al., 2020). This regulator may also affect the expression of additional genes involved in galactose metabolism during growth on d-galactose, and shows that the PCP and d-galactose metabolism are co-regulated (Wu et al., 2020). In *A. niger* and *T. reesei*, the genes involved in d-galactose metabolism showed high expression in response to l-arabinose, d-xylose, and d-galacturonic acid, likely indicating shared utilization of certain genes between these pathways (Fig. 2). Although this also appears to be the case in *N. crassa*, it is not evident at the transcriptome level.

In addition, we tested the growth of seven single deletion mutants, including NCU04442 (*aep-3*), NCU08384 (*xr*), NCU00643 (*ard-1*), NCU00891 (*xyd-1*), NCU04460 (*gpu-1*), NCU08516 (*aep-1*) and NCU01905 (*sodh-1*). Deletion of *xyd-1* and *ard-1* showed a strong reduction of growth on d-galactose (Fig. 7), suggesting that both dehydrogenases are contributing to the oxidoreductive pathway of d-galactose. In addition, more minor growth defects on d-galactose were observed for the deletion of *aep-1*, and *gpu-1* compared to the wild type. NCU04442 (Δ*aep-3*) had an opposite phenotype in the two mating types, preventing us to draw a clear conclusion on its role in this pathway, despite that it is clearly an orthologs of the *A. niger* gene and is therefore predicted to be part of the Leloir pathway.

Deletion of *xr* resulted in reduced growth on d-galactose (Fig. 7), similar to what was observed in *T. reesei* (Seiboth et al., 2007) but different from the case in *A. niger*, in which *xyrB*, and not *xyrA* (the ortholog of *xr*), is involved in the d-galactose oxido-reductive pathway (Chroumpi et al., 2022; Mojzita et al., 2012). Deletion of NCU01905 (*sodh-1*) did not reduce (but rather improved) growth on d-galactose, compared to the reference strain (Fig. 7), questioning its role in the d-galactose oxidoreductive pathway. This gene is one of two predicted orthologs of *A. niger iddA* (previously called *gluE*), which is involved in the d-glucuronic acid pathway (Kuivanen et al., 2016) and a deletion in this gene also did not affect growth of *A. niger* on d-galactose, d-galactitol or sorbitol (Müller, 2024).

Overall, blocking the Leloir pathway did not result in a complete growth arrest on d-galactose, whereas deletion of the d-galactose oxidoreductive pathway genes almost abolished growth on d-galactose in *N. crassa*. This suggests that the GORP may be the dominant pathway of d-galactose metabolism in *N. crassa* (Fig. 7), which is the opposite of the situation in *A. niger* (Chroumpi et al., 2022). In *A. niger*, growth was completely abolished in the strains homologous to the Δ*aep-3* and Δ*gpu-1* strains but was only slightly reduced in the strains homologous to the Δ*xyd-1* and Δ*ard-1* strains (Chroumpi et al., 2022).

### 3.5 Glycolysis, the tricarboxylic acid (TCA) cycle, and glyoxylate cycle

Glycolysis, the tricarboxylic acid (TCA) cycle, and the glyoxylate cycle are central metabolic pathways involved in energy generation and carbon metabolism (Chaudhry and Varacallo, 2018; Cronan Jr and Laporte, 2005; Walsh and Koshland, 1984). As expected, almost all genes involved in these three pathways were detected in *N. crassa* (Suppl. Table S2). The expression levels of almost all glycolytic genes were high during growth on all carbon sources, including d-fructose, except for one predicted triphosphate isomerase (NCU10106, *emp-19*) and three other predicted glycolytic genes (*eno-1* (NCU01870), *emp-11* (NCU09489), and NCU09470, detailed in Suppl. Table S3). Glycolysis was highly conserved among *A. niger, T. reesei* and *N. crassa* with respect to the presence of the main genes. Median expression values were comparable across species (Fig. S1). Globally, there appears to be no overall change of genes involved in glycolysis or the TCA cycle on different carbon sources.

Expression analysis of genes involved in the TCA and glyoxylate cycles revealed that nearly all of them were highly expressed on all carbon sources (Suppl. Table S3) in *N. crassa*. Comparing the three fungi revealed two notable differences. Firstly, while two aconitate hydratase-encoding genes were predicted in *A. niger, T. reesei*, and *N. crassa,* one of the *N. crassa* genes, *tca-3* (NCU02366) was previously identified as part of a complex of predicted TCA cycle genes (Keeping et al., 2011), while the other (NCU04280, *acu-18*) showed very minimal expression. Secondly, one gene copy predicted to encode oxaloacetate acetyl hydrolase (NCU00187, *mig-11*) was detected in *N. crassa* but it was lowly expressed, which is also the case in *T. reesei*.

### 3.6 Pentose phosphate pathway (PPP)

The PPP provides an alternative route for glucose oxidation that is primarily focused on anabolic reactions (Caspi et al., 2013). The PPP fulfills two important anabolic roles: the production of NADPH, a major source of reducing equivalents for biosynthetic reactions, and the synthesis of C4, C5, and C7 sugars. Additionally, it generates ribose 5-phosphate, a critical intermediate for nucleotide and nucleic acid synthesis. The PPP is interconnected with the catabolism of d-ribulose, d-ribose, and d-xylulose, and it produces several glycolytic intermediates, such as glucose 6-phosphate, d-fructose 6-phosphate, d-glyceraldehyde3-phosphate, and NADPH, which are essential components of glycolysis (Fig. 1).

The PPP is conserved across *N. crassa, A. niger*, and *T. reesei*, with all core enzymatic steps encoded in their genomes. Minimal differences among the three species were also observed, primarily restricted to variations in gene copy number (Fig. 3). At the transcriptome level, the expression of predicted PPP genes has no specific carbon source preference, with almost all genes globally expressed (Fig. S1). In addition, several genes exhibited low expression in *N. crassa*, such as those encoding a predicted ribose-5-phosphate isomerase (*rpi-1*; NCU10107), although the promoter of *rpi-1* is bound by the transcription factor XLR-1, which is required for hemicellulose and xylose utilization (Craig, 2015), and predicted transaldolase (NCU06142). Similar in *T. reesei*, the homologous gene encoding TAL also was lowly expressed (Suppl. Table S3). Several additional genes predicted to encode PPP enzymes in *N. crassa* are regulated by XLR-1, including *ppm-8* (NCU01328), *ppm-10* (NCU02136), and *hpd-3* (NCU10110), whose promoter is bound by XLR-1 (Craig, 2015; Sun et al., 2012). However, overall, genes involved in PPP were stably expressed among the three species. This overall conservation, coupled with selectively low expression of certain genes, suggests that while the PPP is structurally intact in all three species, its activity may be modulated in a species-specific manner depending on metabolic demand.

### 3.7 Glycerol pathway (GlycP)

Glycerol, a widely occurring organic compound in nature as well as derivable from the GalAP, serves as a carbon and energy source for many fungi (Klein et al., 2017). Before entering glycolysis, glycerol undergoes conversion into dihydroxyacetone phosphate. The process from glycerol to glycerol 3-phosphate involves the action of glycerol kinase (GLC) (Courtright, 1975) and glycerol 1-phosphatase (GPP). The conversion of glycerol 3-phosphate to dihydroxyacetone phosphate, which enters glycolysis, is mediated by two distinct glycerol 3-phosphate dehydrogenases (GFD). Additionally, glycerol can be converted to dihydroxyacetone by glycerol dehydrogenase (GLD) and subsequently to dihydroxyacetone phosphate by dihydroxyacetone kinase (DAK) (Hondmann et al., 1991).

*N. crassa* possessed a smaller set of genes involved in the GlycP compared with *A. niger*, while its gene repertoire is comparable to that of *T. reesei* (Fig. 3). This similarity with *T. reesei* may indicate a conserved glycerol metabolic capacity among Sordariomycetes. In addition, the genome of *N. crassa* was predicted to encode one copy of each enzyme involved in this pathway, except for the last step with two candidate genes predicted to encode DAK (Suppl. Table S2). Several of these genes have been previously identified in *N. crassa. gcy-1* (NCU04923) and *glp-2* (NCU05454) were shown to have glycerol dehydrogenase activity (Denor and Courtright, 1982; Richter and Hummel, 2011). In addition, transcriptome analysis revealed overall similarity in gene expression patterns of the genes related to this metabolism among the three species (Fig. S1).

### 3.8 **D**-Mannose pathway (ManP)

D-Mannose serves as the structural backbone for the polysaccharide mannan and galactomannan, which is a significant component of hemicellulose found in plant cell walls (Moreira and Filho, 2008). Inside the cell, d-mannose is phosphorylated by hexokinase (HXK) to generate d-mannose 6-phosphate. This compound can be metabolized in two ways: it can be catabolized by mannose 6-phosphate isomerase (PMI) to produce d-fructose 6-phosphate, which then enters glycolysis, or it can undergo sequential enzymatic reactions involving phosphomannomutase (PMM) and mannose-1-phosphate guanylyltransferase (MGT) to be converted into GDP-α-d-mannose (Li et al., 2022).

In general, all reactions of the ManP have at least one gene identified in *N. crassa*, which is similar to the cases in *A. niger* and *T. reesei*, but closer to the situation in *T. reesei* (Fig. 3). However, no distinct mannose-responsive expression was detected for these genes (Suppl. Fig. S3) and deletion of *emp-1* (NCU02542), a predicted hexokinase, did not show reduced growth on d-mannose, suggesting that other genes may be involved in this pathway in *N. crassa* (Fig. S3).

### 3.9 **D**-Glucuronic acid pathway (GluAP)

D-Glucuronic acid is a biomass component occurring, e.g., in the plant cell wall polysaccharide glucuronoxylan and in the algal polysaccharide ulvan (Lahaye and Robic, 2007). A pathway for d-glucuronic acid metabolism has been described for *A. niger* (Kuivanen and Richard, 2018) (Fig. 1). In this pathway, d-glucuronic acid is first reduced to l-gulonate by at least two glucuronate/galacturonate reductases, GAR (Kuivanen et al., 2016; Martens-Uzunova and Schaap, 2008), which are also involved in the fungal d-galacturonic acid pathway (Kuivanen et al., 2016). The gene encoding the enzyme for the oxidation of l-gulonate to 2-keto-l-gulonate has not yet been identified. In the third step, 2-keto-l-gulonate is reduced to l-idonate by two different enzymes, NADH dependent 2-keto-l-gulonate reductase GurA (previously named GluC) and NADPH dependent 2-keto-l-gulonate reductase GurB (previously named GluD) (Kuivanen et al., 2017, 2016). The next step is an oxidation of l-idonate to 5-keto-d-gluconate by the action of l-idonate 5-dehydrogenase IddA (previously named GluE). The 5-keto-d-gluconate is subsequently reduced to d-gluconate by the action of NADPH-requiring 5-keto-d-gluconate reductase KgrA (previously named GluF).

*N. crassa* contains predicted orthologs of all the *A. niger* genes, except *gurA*, but three of the genes (NCU07090, NCU01078, and NCU07242) were lowly expressed (Suppl. Table S3). In addition, several of the genes (e.g., NCU09533 and NCU01906) predicted to play a role in the GluAP are expressed at higher levels in response to other carbon sources and are regulated by PDR-1, PDR-2, or XLR-1, transcription factors required for pectin or xylan degradation (Thieme et al., 2017; Wu et al., 2020) (Suppl. Table S3). These genes may therefore have a role in a different pathway, as neither the *N. crassa* reference strain nor any of the mutants for the genes of this pathway that we tested were able to grow on d-glucuronic acid (data not shown), which could be either due to the low expression of the pathway or another defect, such as lack of a d-glucuronic acid transporter (currently not identified in any fungus).

## 4 Conclusions

Overall, we provide a strongly improved sugar metabolic model for *N. crassa* combining ortholog data with functional annotations of different databases. Major sugar metabolic pathways were predicted as complete in *N. crassa*, while absence of specific genes was observed for the GORP and GlaAP. Compared to *A. niger* and *T. reesei*, several differences and similarities were observed at both the genome and transcriptome levels. In terms of the gene content involved in sugar metabolism, *N. crassa* and *T. reesei* exhibit greater similarity, likely reflecting their closer phylogenetic relationship as members of the Sordariomycetes. As observed in *N. crassa* and *T. reesei*, the number of genes predicted to be related to multiple reactions in glycolysis, PCP, RhaP, and d-galactose metabolism is identical (Fig. 3). Similar patterns were also observed in other pathways, where certain reactions are represented by the same number of predicted orthologs. The difference with *A. niger* is more obvious, as *A. niger* often contains larger number of paralogs of the pathway genes that are often expressed under relevant conditions. In *A. niger*, single deletion strains have no or only a partial phenotype (e.g., (Alazi et al., 2017; Chroumpi et al., 2022, 2021), while many of the *N. crassa* single deletions strains had significant or full phenotypes. This shows that in *N. crassa* often a single enzyme is mainly responsible for a pathway step, while this is shared between multiple enzymes in *A. niger*, and suggests an overall lower gene redundancy in *N. crassa* than in *A. niger* for genes involved in sugar metabolic pathways.

Taken together, the majority of metabolic pathways are highly conserved among the three species analyzed. Moreover, some of these genes are frequently present in single copies, underscoring their evolutionary stability. Representative examples include *gak-1* and *gpu-1* from d-galactose metabolism, and several PCP-related genes, such as *xr, gcy-3, cem-6* and *ard-1*, as well as the entire complement of genes involved in the RhaP (Suppl. Table S2). At the transcriptomic level, the expression profiling of sugar metabolic genes revealed both conserved and divergent regulatory patterns. A consistent feature across the three species is that genes involved in the GalAP and RhaP exhibit strong sugar-induced expression, suggesting a conserved transcriptional response. In contrast, PCP genes displayed species-specific regulation, being sugar-induced in *A. niger* and *T. reesei* but not in *N. crassa* (Fig. 4). Genes involved in d-galactose metabolism also displayed a diverse transcriptional response. Interestingly, for several gene deletions the phenotype differed depending on the mating type in which it was created. The reason for this is currently unclear, but warrants further study.

## Funding sources

JL was supported by NWO NGF‐AiNedXS program NGF.1609.242.042 to JL. MP was supported by NWO NGF‐AiNedXS program NGF.1609.241.001 to MP. This study is based upon work supported by the US National Science Foundation Graduate Research Fellowship to Ruby Schnirman under Grant No. DGE-2139899. This work was partially supported by a grant from the US National Institute of General Medical Sciences of the National Institutes of Health under award number R35GM150926 to L.B.H.

## Data availability

Transcriptome data was obtained from previous studies:

• *N. crassa*: JGI project ID 1053,799 and 1419,356. Raw data can be found on the JGI Genome Portal (https://genome.jgi.doe.gov/portal/NeucrasugarsSet2/NeucrasugarsSet2.info.html) (Wu et al., 2020) and (https://genome.jgi.doe.gov/portal/NeucraEProfiling_211_FD/NeucraEProfiling_211_FD.info.html) (unpublished data).
• *A. niger*: Sequence Read Archive at NCBI under the accession numbers: SRP448993, SRP449003–SRP449007, SRP449023, SRP449039, SRP449049, SRP449062, SRP449079–SRP449081, SRP449083–SRP449085, SRP449089, SRP449068–SRP449070, SRP449098, SRP449125, SRP449138, SRP449141, SRP449142, SRP449151, and SRP449193 (Li et al., 2023)
• *T. reesei*: Sequence Read Archive at NCBI under the accession numbers: SRP378720-SRP378745 (Li et al., 2022)

## CRediT authorship contribution statement

**Jiajia Li:** Conceptualization, Formal analysis, Funding acquisition, Investigation, Supervision, Writing – original draft. **Mao Peng:** Supervision, Writing – review & editing. **Bo Baas:** Formal analysis, Investigation. **Ruby E. Schnirman:** Formal analysis, Investigation, Writing – review & editing. **Lori B. Huberman:** Conceptualization, Funding acquisition, Supervision, Writing – review & editing. **Ronald P. de Vries:** Conceptualization, Funding acquisition, Supervision, Writing – review & editing.

## Declaration of competing interest

The authors declare that they have no known competing financial interests or personal relationships that could have appeared to influence the work reported in this paper.

## Appendix Supplementary materials

Figure S1. Comparison of the biomass production (mycelial dry weight) of the different strains grown on d-glucose. A. Strains in the mating type A (*mat A*) background. B. Strains in the mating type a (*mat a*) background. WT = wild type. The other strains are deletions for the gene indicated by the gene number.

Figure S2. Change in transcription level of the genes assigned to metabolic pathways under different carbon sources in *Aspergillus niger, Neurospora crassa* and *Trichoderma reesei*. Boxplots showing the change of expression of genes involved in different metabolic pathways depending on the carbon source used.

Figure S3. Growth of *N. crassa* on d-mannose. The indicated *N. crassa* strains were inoculated into media containing d-mannose as the sole carbon source and incubated at 28 °C.

Image, application 1

Image, application 2

Image, application 3

Image, application 4

Image, application 5

Image, application 6

Image, application 7

## References

[bib0001] Aguilar-Pontes M.V. (2018). The gold-standard genome of *Aspergillus niger* NRRL 3 enables a detailed view of the diversity of sugar catabolism in fungi. Stud. Mycol..

[bib0002] Akel E. (2009). Molecular regulation of arabinan and L-arabinose metabolism in *Hypocrea jecorina* (Trichoderma reesei). Eukaryot. Cell.

[bib0003] Alazi E. (2017). The pathway intermediate 2-keto-3-deoxy-L-galactonate mediates the induction of genes involved in D-galacturonic acid utilization in *Aspergillus niger*. FEBS Lett..

[bib0004] Alazi E. (2016). The transcriptional activator GaaR of *Aspergillus niger* is required for release and utilization of D-galacturonic acid from pectin. FEBS Lett..

[bib0005] Ashburner M. (2000). Gene ontology: tool for the unification of biology. Nat. Genet..

[bib0006] Bae B. (2010). Structure and engineering of L-arabinitol 4-dehydrogenase from *Neurospora crassa*. J. Mol. Biol..

[bib0007] Benz J.P. (2014). A comparative systems analysis of polysaccharide-elicited responses in *Neurospora crassa* reveals carbon source-specific cellular adaptations. Mol. Microbiol..

[bib0008] Brody S., Tatum E. (1967). Phosphoglucomutase mutants and morphological changes in *Neurospora crassa*. Proc. Natl. Acad. Sci. U.S.A..

[bib0009] Caspi R. (2014). The MetaCyc database of metabolic pathways and enzymes and the BioCyc collection of Pathway/Genome databases. Nucleic. Acids. Res..

[bib0010] Caspi R. (2013). The challenge of constructing, classifying, and representing metabolic pathways. FEMS Microbiol. Lett..

[bib0011] Chaudhry, R., Varacallo, M., 2018. Biochemistry, glycolysis. StatPearls Publishing, Treas. Island. 29493928

[bib0012] Chroumpi T. (2020). Identification of a gene encoding the last step of the L-rhamnose catabolic pathway in *Aspergillus niger* revealed the inducer of the pathway regulator. Microbiol. Res..

[bib0013] Chroumpi T. (2022). Detailed analysis of the D-galactose catabolic pathways in *Aspergillus niger* reveals complexity at both metabolic and regulatory level. Fungal. Genet. Biol..

[bib0014] Chroumpi T. (2021). Revisiting a 'simple' fungal metabolic pathway reveals redundancy, complexity and diversity. Microb. Biotechnol..

[bib0015] Colot H.V. (2006). A high-throughput gene knockout procedure for *Neurospora* reveals functions for multiple transcription factors. Proc. Natl. Acad. Sci. U.S.A..

[bib0016] Coradetti S.T. (2012). Conserved and essential transcription factors for cellulase gene expression in ascomycete fungi. Proc. Natl. Acad. Sci. U.S.A..

[bib0017] Courtright J.B. (1975). Intracellular localization and properties of glycerokinase and glycerophosphate dehydrogenase in *Neurospora crassa*. Arch. Biochem. Biophys..

[bib0018] Craig J.P. (2015). Direct target network of the *Neurospora crassa* plant cell wall deconstruction regulators CLR-1, CLR-2, and XLR-1. mBio.

[bib0019] Cronan J.E., Laporte D. (2005). Tricarboxylic acid cycle and glyoxylate bypass. EcoSal. Plus..

[bib0020] Davis R.H., Perkins D.D. (2002). *Neurospora*: a model of model microbes. Nat. Rev. Genet..

[bib0021] de Vries R.P., Li J. (2026). Do we need a more structured system for gene names and pathways now that omics studies revealed a higher complexity of fungal carbon metabolism?. Fung. Biol. Rev..

[bib0022] Denor P.F., Courtright J.B. (1982). Genetic and enzymatic characterization of the inducible glycerol dissimilatory system of *Neurospora crassa*. J. Bacteriol..

[bib0023] Dreyfuss J.M. (2013). Reconstruction and validation of a genome-scale metabolic model for the filamentous fungus *Neurospora crassa* using FARM. PLoS Comp. Biol..

[bib0024] Emms D.M., Kelly S. (2019). OrthoFinder: phylogenetic orthology inference for comparative genomics. Genome Biol..

[bib0025] Galagan J.E. (2003). The genome sequence of the filamentous fungus *Neurospora crassa*. Nature.

[bib0026] Grigoriev I.V. (2014). MycoCosm portal: gearing up for 1000 fungal genomes. Nucleic. Acids. Res..

[bib0027] Gruben B.S. (2014). *Aspergillus niger* RhaR, a regulator involved in L-rhamnose release and catabolism. Appl. Microbiol. Biotechnol..

[bib0028] Hondmann D.H. (1991). Glycerol catabolism in *Aspergillus nidulans*. Microbiology.

[bib0029] Hunter S. (2009). InterPro: the integrative protein signature database. Nucleic. Acids. Res..

[bib0030] Irmak S., Tumuluru J.S. (2017). Biomass As Raw Material For Production of High-Value. Biomass Volume Estimation and Valorization for Energy.

[bib0031] Kanehisa M. (2025). KEGG: biological systems database as a model of the real world. Nucleic. Acids. Res..

[bib0032] Kanehisa M., Goto S. (2000). KEGG: kyoto encyclopedia of genes and genomes. Nucleic. Acids. Res..

[bib0033] Karp P.D. (2021). Pathway Tools version 23.0 update: software for pathway/genome informatics and systems biology. Brief. Bioinformat..

[bib0034] Karp P.D. (2002). The pathway tools software. Bioinformatics.

[bib0035] Keeping A. (2011). Gel-based mass spectrometric and computational approaches to the mitochondrial proteome of *Neurospora*. Fungal. Genet. Biol..

[bib0036] Khosravi C. (2017). In vivo functional analysis of L-rhamnose metabolic pathway in *Aspergillus niger*: a tool to identify the potential inducer of RhaR. BMC. Microbiol..

[bib0037] Klein M. (2017). Glycerol metabolism and transport in yeast and fungi: established knowledge and ambiguities. Environ. Microbiol..

[bib0038] Kuivanen J. (2017). Clustered genes encoding 2-keto-L-gulonate reductase and L-idonate 5-dehydrogenase in the novel fungal D-glucuronic acid pathway. Front. Microbiol..

[bib0039] Kuivanen J., Richard P. (2018). NADPH-dependent 5-keto-D-gluconate reductase is a part of the fungal pathway for D-glucuronate catabolism. FEBS Lett..

[bib0040] Kuivanen J. (2016). A novel pathway for fungal D-glucuronate catabolism contains an L-idonate forming 2-keto-L-gulonate reductase. Sci. Rep..

[bib0041] Kulikova M. (2022). Plant biomass as a raw material for producing basic organic sysnthesis products. Chem. Technol. Fuels Oils..

[bib0042] Kuorelahti S. (2006). l-galactonate dehydratase is part of the fungal path for D-galacturonic acid catabolism. Mol. Microbiol..

[bib0043] Lahaye M., Robic A. (2007). Structure and functional properties of ulvan, a polysaccharide from green seaweeds. Biomacromol.

[bib0044] Lamb T.M. (2012). The *Neurospora crassa* OS MAPK pathway-activated transcription factor ASL-1 contributes to circadian rhythms in pathway responsive clock-controlled genes. Fungal. Genet. Biol..

[bib0045] Li J. (2022). The sugar metabolic model of *Aspergillus niger* can only be reliably transferred to fungi of its phylum. J. Fungi..

[bib0046] Li J. (2014). Transcriptional comparison of the filamentous fungus neurospora crassa growing on three major monosaccharides D-glucose, D-xylose and L-arabinose. Biotechnol. Biofuels..

[bib0047] Li J. (2023). Comparative genomics and transcriptomics analyses reveal divergent plant biomass-degrading strategies in fungi. J. Fungi..

[bib0048] Li W.-C. (2017). *Trichoderma reesei* complete genome sequence, repeat-induced point mutation, and partitioning of CAZyme gene clusters. Biotechnol. Biofuels..

[bib0049] Liepins J. (2006). Enzymes for the NADPH-dependent reduction of dihydroxyacetone and D-glyceraldehyde and L-glyceraldehyde in the mould *Hypocrea jecorina*. FEBS. J..

[bib0050] Martens-Uzunova E.S., Schaap P.J. (2008). An evolutionary conserved D-galacturonic acid metabolic pathway operates across filamentous fungi capable of pectin degradation. Fungal. Genet. Biol..

[bib0051] Martinez D. (2008). Genome sequencing and analysis of the biomass-degrading fungus *Trichoderma reesei* (syn. Hypocrea jecorina). Nat. Biotechnol..

[bib0052] McCluskey K. (2010). The fungal genetics stock center: a repository for 50 years of fungal genetics research. J. Biosci..

[bib0053] Mistry J. (2021). Pfam: the protein families database in 2021. Nucleic. Acids. Res..

[bib0054] Mojzita D. (2012). l-xylo-3-hexulose reductase is the missing link in the oxidoreductive pathway for D-galactose catabolism in filamentous fungi. J. Biol. Chem..

[bib0055] Mojzita D. (2010). Identification of an l-arabinose reductase gene in *Aspergillus niger* and its role in L-arabinose catabolism. J. Biol. Chem..

[bib0056] Moreira L., Filho E. (2008). An overview of mannan structure and mannan-degrading enzyme systems. Appl. Microbiol. Biotechnol..

[bib0057] Müller A. (2024).

[bib0058] Paysan-Lafosse T. (2023). InterPro in 2022. Nucleic. Acids. Res..

[bib0059] Protzko R.J. (2019). Genomewide and enzymatic analysis reveals efficient D-galacturonic acid metabolism in the basidiomycete yeast *Rhodosporidium toruloides*. mSystems.

[bib0060] Radford A. (2004). Metabolic highways of *Neurospora crassa* revisited. Adv. Genet..

[bib0061] Richard P., Hilditch S. (2009). D-galacturonic acid catabolism in microorganisms and its biotechnological relevance. Appl. Microbiol. Biotechnol..

[bib0062] Richter N., Hummel W. (2011). Biochemical characterisation of a NADPH-dependent carbonyl reductase from Neurospora crassa reducing α-and β-keto esters. Enzyme Microb. Technol..

[bib0063] Ritchie M.E. (2015). Limma powers differential expression analyses for RNA-sequencing and microarray studies. Nucleic. Acids. Res..

[bib0064] Samal A. (2017). Network reconstruction and systems analysis of plant cell wall deconstruction by *Neurospora crassa*. Biotechnol. Biofuels..

[bib0065] Seiboth B. (2007). The D-xylose reductase of *Hypocrea jecorin*a is the major aldose reductase in pentose and D-galactose catabolism and necessary for β-galactosidase and cellulase induction by lactose. Mol. Microbiol..

[bib0066] Seiboth B. (2003). D-xylose metabolism in *Hypocrea jecorina*: loss of the xylitol dehydrogenase step can be partially compensated for by lad1-encoded L-arabinitol-4-dehydrogenase. Eukaryot. Cell.

[bib0067] Seiboth B., Metz B. (2011). Fungal arabinan and L-arabinose metabolism. Appl. Microbiol. Biotechnol..

[bib0068] Seiler S., Plamann M. (2003). The genetic basis of cellular morphogenesis in the filamentous fungus *Neurospora crassa*. Mol. Biol. Cell.

[bib0069] Sonnhammer E.L. (1997). Pfam: a comprehensive database of protein domain families based on seed alignments. Proteins: Struct. Funct. Bioinform..

[bib0070] Sullivan R., Zhao H. (2007). Cloning, characterization, and mutational analysis of a highly active and stable L-arabinitol 4-dehydrogenase from *Neurospora crassa*. Appl. Microbiol. Biotechnol..

[bib0071] Sun J. (2012). Deciphering transcriptional regulatory mechanisms associated with hemicellulose degradation in *Neurospora crassa*. Eukaryot. Cell.

[bib0072] Thieme N. (2017). The transcription factor PDR-1 is a multi-functional regulator and key component of pectin deconstruction and catabolism in *Neurospora crassa*. Biotechnol. Biofuels..

[bib0073] Tian C. (2009). Systems analysis of plant cell wall degradation by the model filamentous fungus *Neurospora crassa*. Proc. Nat. Acad. Sci. U.S.A..

[bib0074] Vesth T.C. (2018). Investigation of inter-and intraspecies variation through genome sequencing of *Aspergillus* section *Nigri*. Nat. Genet..

[bib0075] Vogel H.J. (1956). A convenient growth medium for *Neurospora*. Microbiol. Genet. Bull..

[bib0076] Walsh K., Koshland D.E. (1984). Determination of flux through the branch point of two metabolic cycles. The tricarboxylic acid cycle and the glyoxylate shunt. J. Biol. Chem..

[bib0077] Watanabe S. (2008). Eukaryotic and bacterial gene clusters related to an alternative pathway of nonphosphorylated L-rhamnose metabolism. J. Biol. Chem..

[bib0078] Witteveen C. (1989). L-arabinose and D-xylose catabolism in *Aspergillus niger*. Microbiology.

[bib0079] Woodyer R. (2005). Heterologous expression, purification, and characterization of a highly active xylose reductase from *Neurospora crassa*. Appl. Environ. Microbiol..

[bib0080] Wu V.W. (2020). The regulatory and transcriptional landscape associated with carbon utilization in a filamentous fungus. Proc. Natl. Acad. Sci. U.S.A..

[bib0081] Zhao X. (1998). The production and properties of a new xylose reductase from fungus: *Neurospora crassa*. Appl. Biochem. Biotechnol..

