# Supplementary material for: The genome-scale sugar metabolic model from Neurospora crassa reveals lower gene redundancy than that of Aspergillus niger
Source: Curr Res Microb Sci. 2026 Apr 15;10:100596. doi: 10.1016/j.crmicr.2026.100596 (PMC13158570; doi:10.1016/j.crmicr.2026.100596)
Supplement: Supplementary file 1 [file mmc1.pdf]

A.

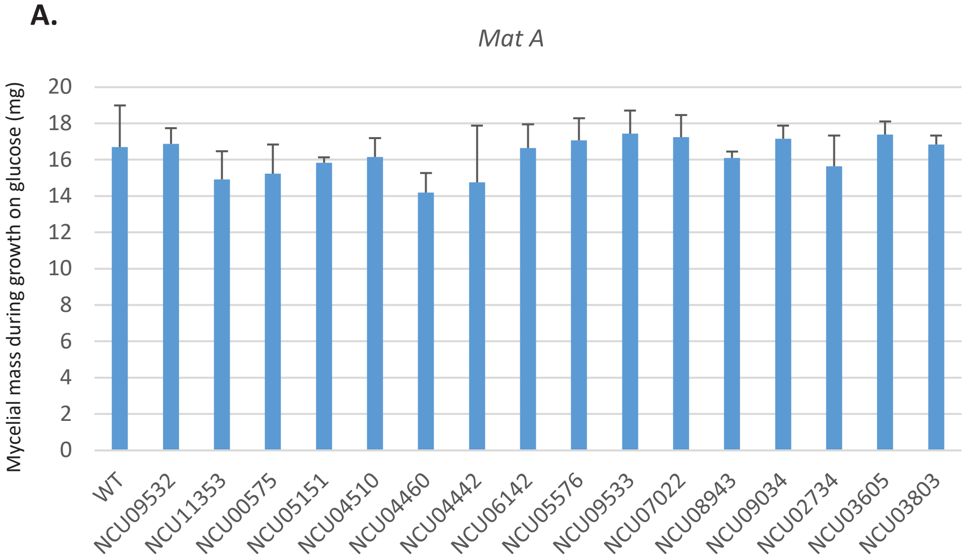

B.

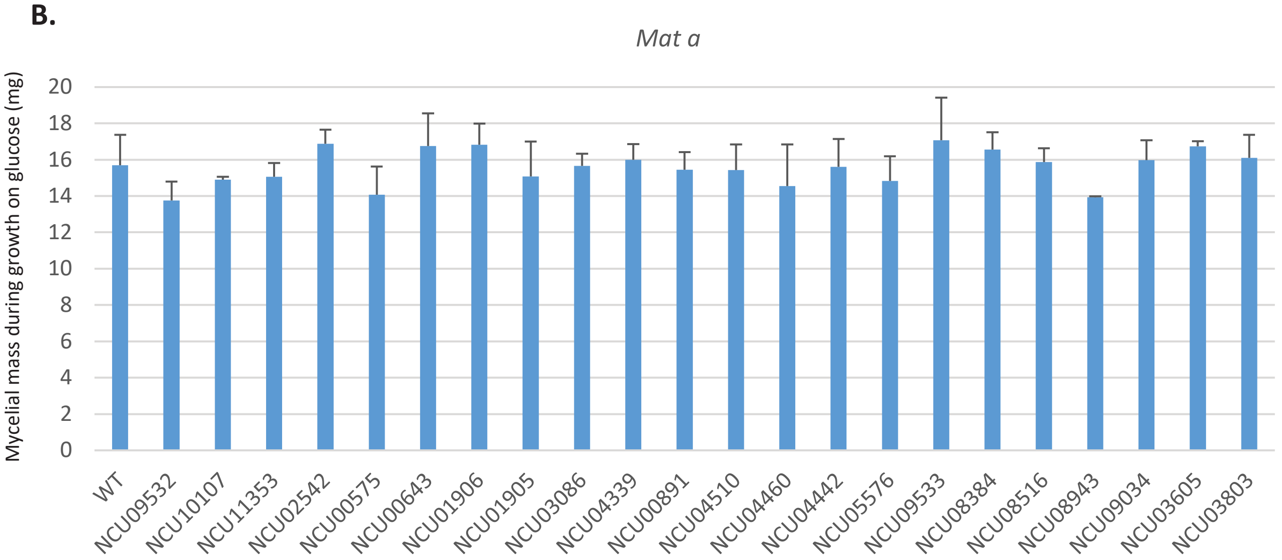

Supplemental Figure S1. Comparison of the biomass production of the different strains grown on D-glucose. A. Strains in the mating type A background. B. Strains in the mating type a background.
