# Supplementary material for: The genome-scale sugar metabolic model from Neurospora crassa reveals lower gene redundancy than that of Aspergillus niger
Source: Curr Res Microb Sci. 2026 Apr 15;10:100596. doi: 10.1016/j.crmicr.2026.100596 (PMC13158570; doi:10.1016/j.crmicr.2026.100596)
Supplement: Supplementary file 2 [file mmc2.pdf]

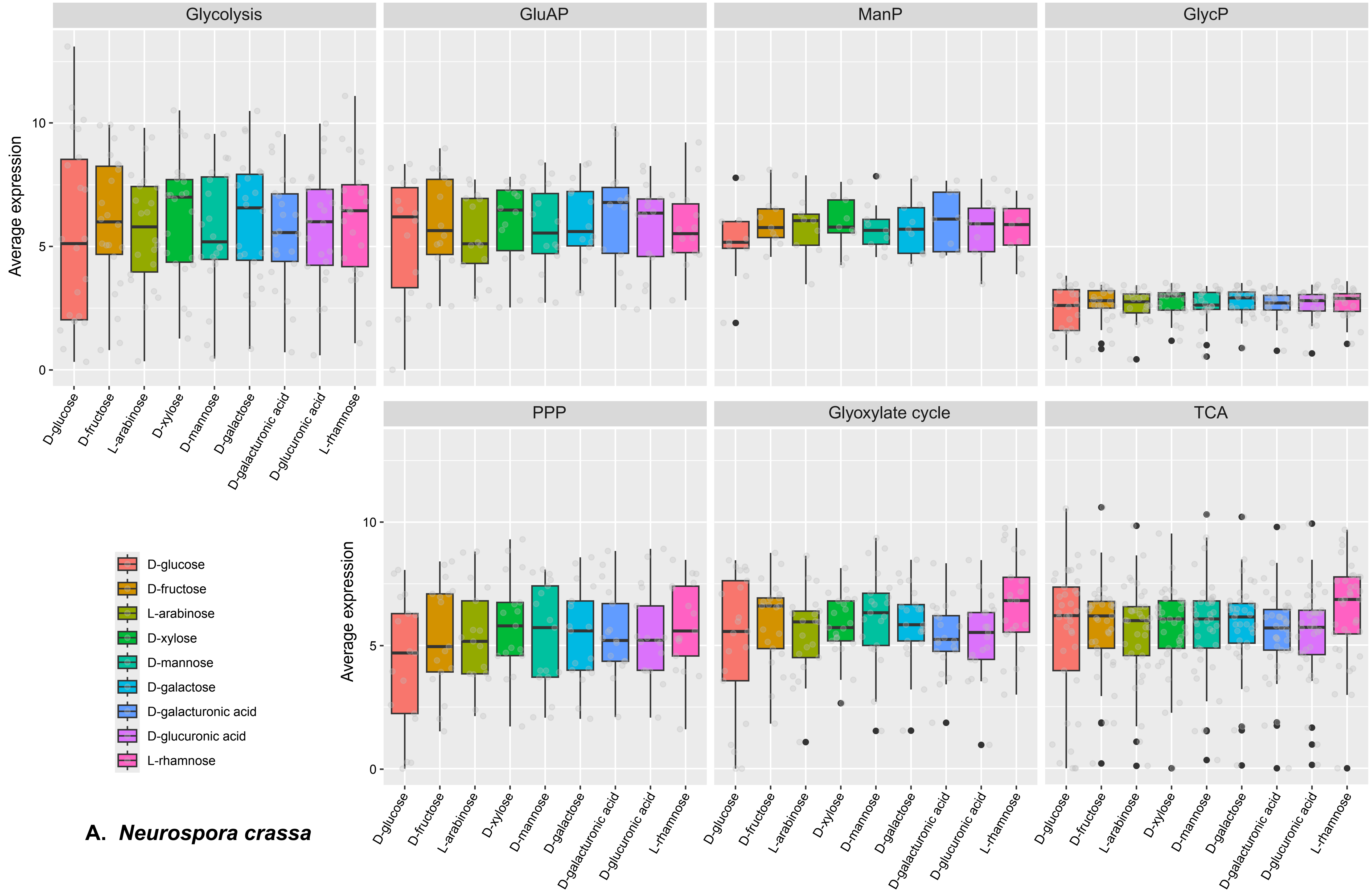

A. *Neurospora crassa*

Supplemental Figure S2. A. Change in transcription level (FPKM) of the genes assigned to metabolic pathways under different carbon sources in ***Neurospora crassa***. Boxplots showing the change of expression of specific genes involved in their corresponding metabolic pathways depending on the carbon source used. Various colors of boxplots indicated different carbon sources.

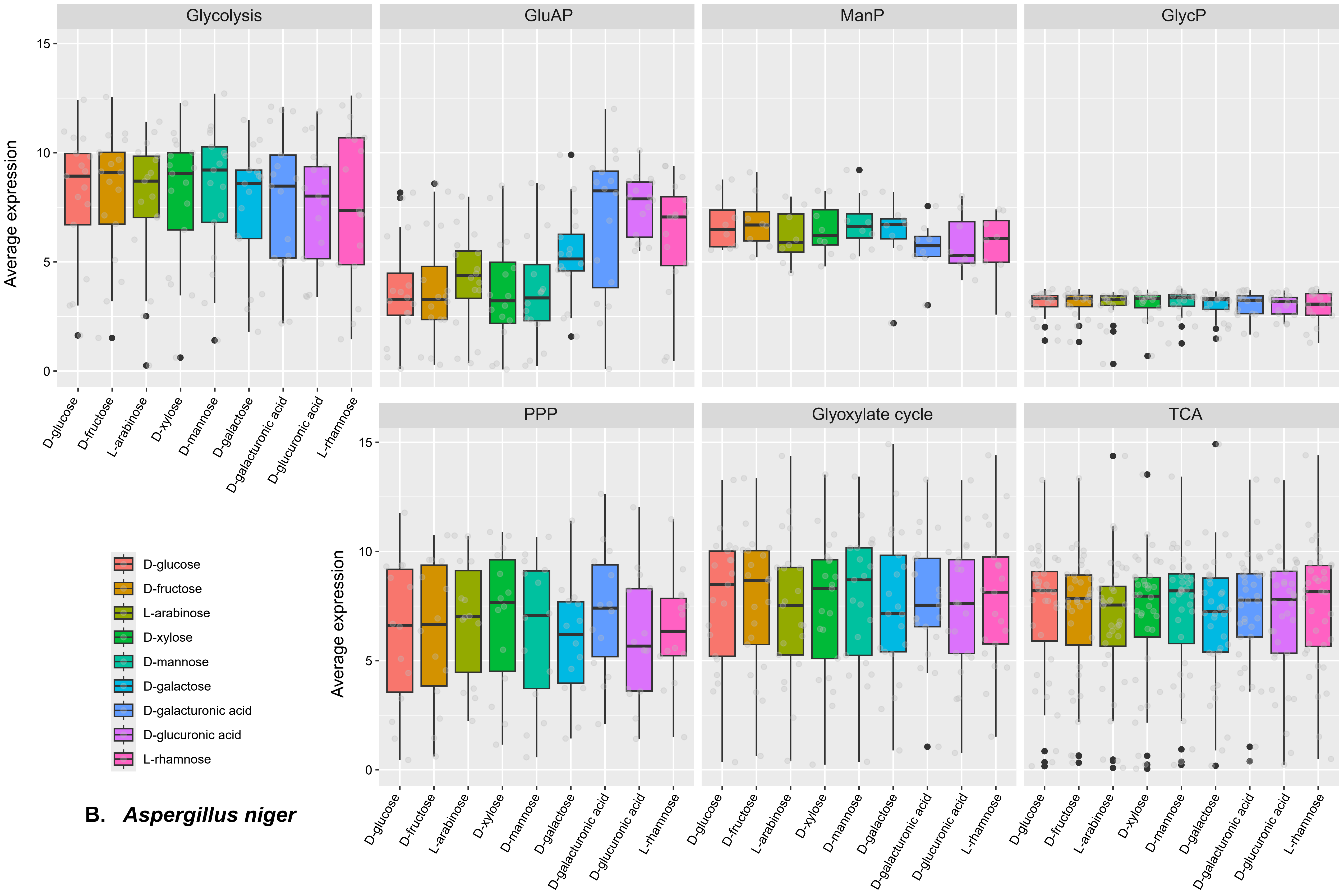

Supplemental Figure S2. B. Change in transcription level (FPKM) of the genes assigned to metabolic pathways under different carbon sources in ***Aspergillus niger***. Boxplots showing the change of expression of specific genes involved in their corresponding metabolic pathways depending on the carbon source used. Various colors of boxplots indicated different carbon sources.

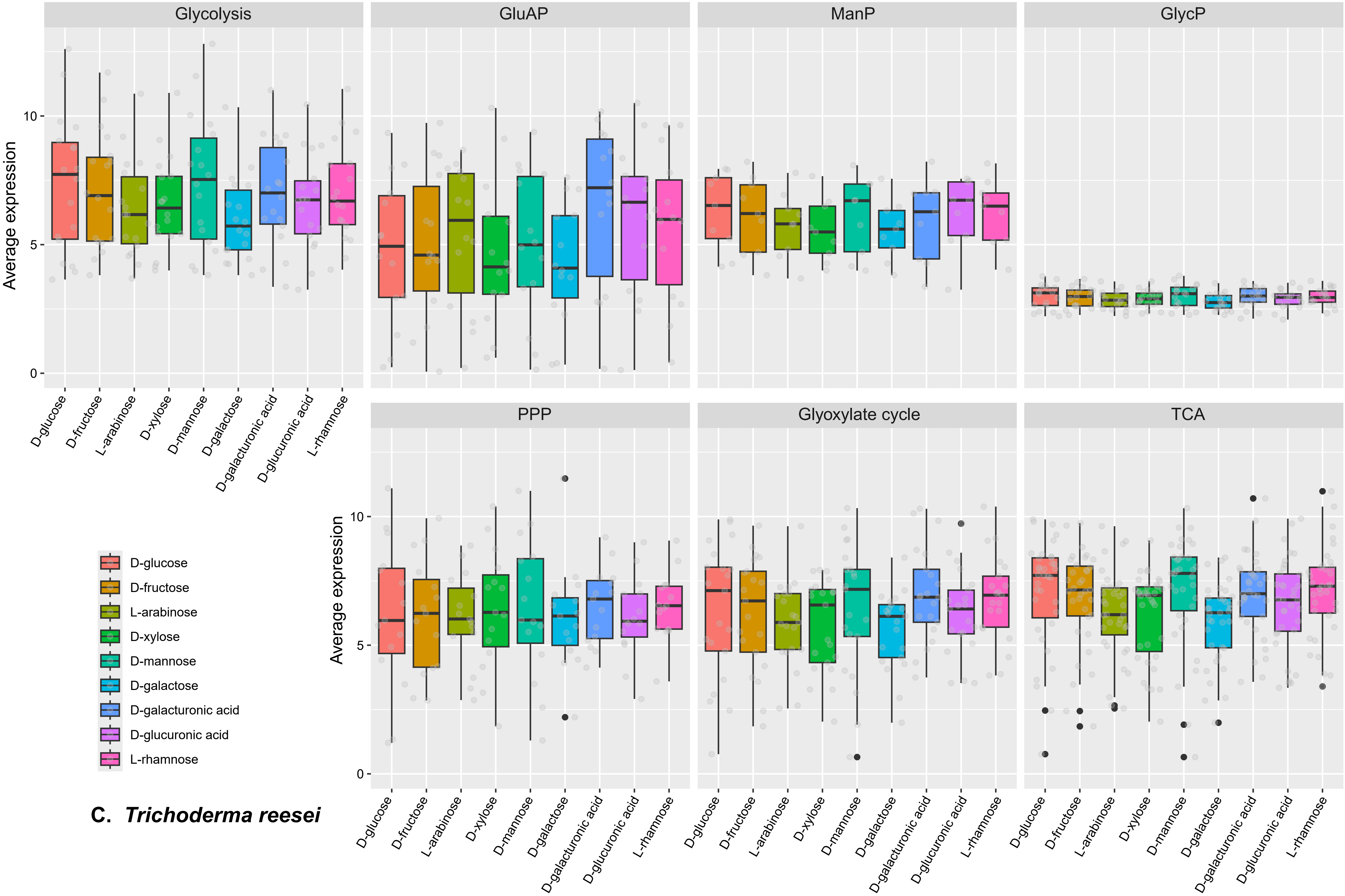

C. *Trichoderma reesei*

Supplemental Figure S2. C. Change in transcription level (FPKM) of the genes assigned to metabolic pathways under different carbon sources in ***Trichoderma reesei***. Boxplots showing the change of expression of specific genes involved in their corresponding metabolic pathways depending on the carbon source used. Various colors of boxplots indicated different carbon sources.
