# Supplementary material for: The genome-scale sugar metabolic model from Neurospora crassa reveals lower gene redundancy than that of Aspergillus niger
Source: Curr Res Microb Sci. 2026 Apr 15;10:100596. doi: 10.1016/j.crmicr.2026.100596 (PMC13158570; doi:10.1016/j.crmicr.2026.100596)
Supplement: Supplementary file 3 [file mmc3.pdf]

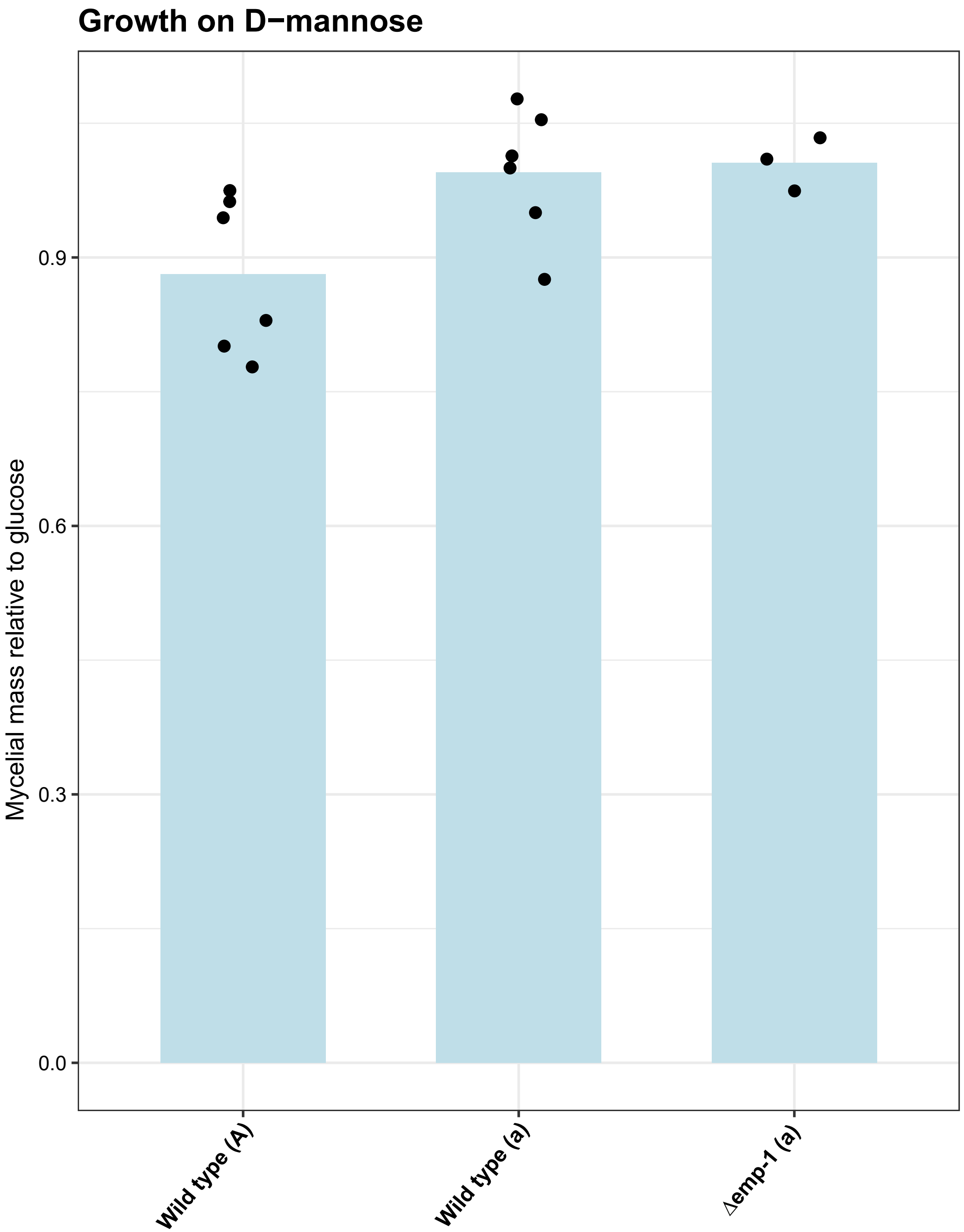

Supplemental Figure S3. Phenotypic analysis of Δemp-1 (NCU02542) during growth on D-mannose in the wild type strains of *N. crassa* and deletion mutants.
