# Supplementary material for: The genome-scale sugar metabolic model from Neurospora crassa reveals lower gene redundancy than that of Aspergillus niger
Source: Curr Res Microb Sci. 2026 Apr 15;10:100596. doi: 10.1016/j.crmicr.2026.100596 (PMC13158570; doi:10.1016/j.crmicr.2026.100596)
Supplement: Supplementary file 5 [file mmc5.pdf]

Supplemental Table S3. Overview of incubation times of the growth on the different carbon sources.

**Gene deleted (NCU number)**	**Mating type**	**Carbon source**	**Length of incubation (days)**	**Number of biological replicates**	**Replicates spread across multiple days (Yes/No)**
Wild type	*mat A*	L-arabinose	3	9	Yes
Wild type	*mat a*	L-arabinose	3	9	Yes
NCU04510	*mat A*	L-arabinose	3	3	No
NCU04510	*mat a*	L-arabinose	3	3	No
NCU08384	*mat a*	L-arabinose	3	3	No
NCU00643	*mat a*	L-arabinose	3	3	No
NCU00891	*mat a*	L-arabinose	3	3	No
NCU08943	*mat A*	L-arabinose	3	3	No
NCU08943	*mat a*	L-arabinose	3	3	No
NCU03803	*mat a*	L-arabinose	3	3	No
NCU01905	*mat a*	L-arabinose	3	3	No
NCU01906	*mat a*	L-arabinose	3	3	No
NCU11353	*mat A*	L-arabinose	3	3	No
NCU11353	*mat a*	L-arabinose	3	3	No
Wild type	*mat A*	D-mannose	2	6	Yes
Wild type	*mat a*	D-mannose	2	6	Yes
NCU02542	*mat a*	D-mannose	2	6	Yes
Wild type	*mat A*	D-xylose	2	18	Yes
Wild type	*mat a*	D-xylose	2	18	Yes
NCU04510	*mat A*	D-xylose	2	6	Yes
NCU04510	*mat a*	D-xylose	2	9	Yes
NCU01905	*mat a*	D-xylose	2	6	Yes
NCU01906	*mat a*	D-xylose	2	3	No
NCU11353	*mat A*	D-xylose	2	3	No
NCU11353	*mat a*	D-xylose	2	3	No
NCU10107	*mat a*	D-xylose	2	3	No
NCU04339	*mat a*	D-xylose	2	3	No
NCU06142	*mat A*	D-xylose	2	3	No
NCU06142	*mat a*	D-xylose	2	3	No
NCU05151	*mat A*	D-xylose	2	3	No
NCU00643	*mat a*	D-xylose	2	3	No
NCU00891	*mat a*	D-xylose	2	3	No
NCU08384	*mat a*	D-xylose	2	3	No
Wild type	*mat A*	D-glucose	2	27	Yes
Wild type	*mat a*	D-glucose	2	27	Yes
NCU04510	*mat A*	D-glucose	2	9	Yes
NCU04510	*mat a*	D-glucose	2	9	Yes
NCU00643	*mat a*	D-glucose	2	12	Yes
NCU08943	*mat A*	D-glucose	2	3	No
NCU08943	*mat a*	D-glucose	2	3	No
NCU03803	*mat A*	D-glucose	2	3	No
NCU03803	*mat a*	D-glucose	2	6	Yes

NCU02542	*mat a*	D-glucose	2	3	No
NCU11353	*mat A*	D-glucose	2	6	Yes
NCU11353	*mat a*	D-glucose	2	6	Yes
NCU10107	*mat a*	D-glucose	2	3	No
NCU04339	*mat a*	D-glucose	2	3	No
NCU06142	*mat A*	D-glucose	2	3	No
NCU06142	*mat a*	D-glucose	2	3	No
NCU05151	*mat A*	D-glucose	2	3	No
NCU01905	*mat a*	D-glucose	2	9	Yes
NCU08384	*mat a*	D-glucose	2	6	Yes
NCU00891	*mat a*	D-glucose	2	12	Yes
NCU04442	*mat A*	D-glucose	2	6	Yes
NCU04442	*mat a*	D-glucose	2	6	Yes
NCU09034	*mat A*	D-glucose	2	6	Yes
NCU09034	*mat a*	D-glucose	2	3	No
NCU09533	*mat A*	D-glucose	2	12	Yes
NCU09533	*mat a*	D-glucose	2	12	Yes
NCU01906	*mat a*	D-glucose	2	9	Yes
NCU09532	*mat A*	D-glucose	2	6	Yes
NCU09532	*mat a*	D-glucose	2	6	Yes
NCU03605	*mat A*	D-glucose	2	6	Yes
NCU03605	*mat a*	D-glucose	2	6	Yes
NCU03086	*mat a*	D-glucose	2	6	Yes
NCU02734	*mat A*	D-glucose	2	3	No
NCU08516	*mat a*	D-glucose	2	6	Yes
NCU04460	*mat A*	D-glucose	2	9	Yes
NCU04460	*mat a*	D-glucose	2	12	Yes
NCU00575	*mat A*	D-glucose	2	9	Yes
NCU00575	*mat a*	D-glucose	2	12	Yes
NCU05576	*mat A*	D-glucose	2	3	No
NCU05576	*mat a*	D-glucose	2	3	No
NCU07022	*mat A*	D-glucose	2	6	Yes
Wild type	*mat A*	D-galactose	8	15	Yes
Wild type	*mat a*	D-galactose	8	18	Yes
NCU08384	*mat a*	D-galactose	8	3	No
NCU04442	*mat A*	D-galactose	8	3	No
NCU04442	*mat a*	D-galactose	8	6	Yes
NCU00643	*mat a*	D-galactose	8	3	No
NCU00891	*mat a*	D-galactose	8	3	No
NCU08516	*mat a*	D-galactose	8	3	No
NCU04460	*mat A*	D-galactose	8	6	Yes
NCU04460	*mat a*	D-galactose	8	9	Yes
NCU00575	*mat A*	D-galactose	8	6	Yes
NCU00575	*mat a*	D-galactose	8	8	Yes
NCU05576	*mat A*	D-galactose	8	3	No
NCU05576	*mat a*	D-galactose	8	3	No
NCU01905	*mat a*	D-galactose	8	3	No

NCU01906	*mat a*	D-galactose	8	3	No
Wild type	*mat A*	L-rhamnose	4	9	Yes
Wild type	*mat a*	L-rhamnose	4	9	Yes
NCU09034	*mat A*	L-rhamnose	4	3	No
NCU09034	*mat a*	L-rhamnose	4	3	No
NCU03605	*mat A*	L-rhamnose	4	3	No
NCU03605	*mat a*	L-rhamnose	4	3	No
NCU03086	*mat a*	L-rhamnose	4	3	No
NCU02734	*mat A*	L-rhamnose	4	3	No
Wild type	*mat A*	D-galacturonic acid	13	6	Yes
Wild type	*mat a*	D-galacturonic acid	13	6	Yes
NCU09533	*mat A*	D-galacturonic acid	13	3	No
NCU09533	*mat a*	D-galacturonic acid	13	3	No
NCU01905	*mat a*	D-galacturonic acid	13	3	No
NCU01906	*mat a*	D-galacturonic acid	13	3	No
NCU09532	*mat A*	D-galacturonic acid	13	3	No
NCU09532	*mat a*	D-galacturonic acid	13	3	No
Wild type	*mat A*	D-glucuronic acid	15	3	No
Wild type	*mat a*	D-glucuronic acid	15	3	No
