# Supplementary material for: The genome-scale sugar metabolic model from Neurospora crassa reveals lower gene redundancy than that of Aspergillus niger
Source: Curr Res Microb Sci. 2026 Apr 15;10:100596. doi: 10.1016/j.crmicr.2026.100596 (PMC13158570; doi:10.1016/j.crmicr.2026.100596)
Supplement: Supplementary file 7 [file mmc7.pdf]

Supplementary Table S1. The corresponding protein IDs of deletion mutations of *N. crassa*

Name	Deleted gene (NCU number)	Genotype	Source
Wild type	N/A	*mat A*	FGSC 2489 (McCluskey et al., 2010)
Wild type	N/A	*mat a*	FGSC 4200 (McCluskey et al., 2010)
Δ*xr*	NCU08384	Δ*xr::hph mat a*	FGSC 20307 (Colot et al., 2006)
Δ*ard-1*	NCU00643	Δ*ard-1::hph mat a*	FGSC 15929 (Colot et al., 2006)
Δ*xyd-1*	NCU00891	Δ*xyd-1::hph mat a*	FGSC 16529 (Colot et al., 2006)
Δ*aep-1*	NCU08516	Δ*aep-1::hph mat a*	FGSC 20323 (Colot et al., 2006)
Δ*aep-3*	NCU04442	Δ*aep-3::hph mat A*	FGSC 16813 (Colot et al., 2006)
Δ*aep-3*	NCU04442	Δ*aep-3::hph mat a*	FGSC 16823 (Colot et al., 2006)
Δ*gpu-1*	NCU04460	Δ*gpu-1::hph mat A*	FGSC 16787 (Colot et al., 2006)
Δ*gpu-1*	NCU04460	Δ*gpu-1::hph mat a*	FGSC 16788 (Colot et al., 2006)
ΔNCU09533	NCU09533	ΔNCU09533*::hph mat A*	FGSC 19857 (Colot et al., 2006)
ΔNCU09533	NCU09533	ΔNCU09533*::hph mat a*	FGSC 19856 (Colot et al., 2006)
Δ*gcy-2*	NCU01906	Δ*gcy-2::hph mat a*	FGSC 13273 (Colot et al., 2006)
Δ*lga-1*	NCU09532	Δ*lga-1::hph mat A*	FGSC 21064 (Colot et al., 2006)
Δ*lga-1*	NCU09532	Δ*lga-1::hph mat a*	FGSC 21065 (Colot et al., 2006)
Δ*lrl-1*	NCU03605	Δ*lrl-1::hph mat A*	FGSC 11879 (Colot et al., 2006)
Δ*lrl-1*	NCU03605	Δ*lrl-1::hph mat a*	FGSC 11878 (Colot et al., 2006)
Δ*lrd-1*	NCU09034	Δ*lrd-1::hph mat A*	FGSC 18453 (Colot et al., 2006)
Δ*lrd-1*	NCU09034	Δ*lrd-1::hph mat a*	FGSC 18452 (Colot et al., 2006)
Δ*cem-6*	NCU08943	Δ*cem-6::hph mat A*	FGSC 18436 (Colot et al., 2006)
Δ*cem-6*	NCU08943	Δ*cem-6::hph mat a*	FGSC 18435 (Colot et al., 2006)
ΔNCU03086	NCU03086	ΔNCU03086*::hph mat a*	FGSC 16334 (Colot et al., 2006)
Δ*emp-1*	NCU02542	Δ*emp-1::hph mat a*	FGSC 11220 (Colot et al., 2006)
Δ*gcy-3*	NCU04510	Δ*gyc-3::hph mat A*	FGSC 16701 (Colot et al., 2006)
Δ*gcy-3*	NCU04510	Δ*gcy-3::hph mat a*	FGSC 16700 (Colot et al., 2006)
Δ*sodh-1*	NCU01905	Δ*sodh-1::hph mat a*	FGSC 13367 (Colot et al., 2006)
Δ*xyk-1*	NCU11353	Δ*xyk-1::hph mat A*	FGSC 23595 (Colot et al., 2006)
Δ*xyk-1*	NCU11353	Δ*xyk-1::hph mat a*	FGSC 23594 (Colot et al., 2006)
Δ*rpi-1*	NCU10107	Δ*rpi-1::hph mat a*	FGSC 21537 (Colot et al., 2006)
Δ*rik-8*	NCU04339	Δ*rik-8::hph mat a*	FGSC 16454 (Colot et al., 2006)
ΔNCU06142	NCU06142	ΔNCU06142*::hph mat A*	FGSC 14312 (Colot et al., 2006)
ΔNCU06142	NCU06142	ΔNCU06142*::hph mat a*	FGSC 14311 (Colot et al., 2006)
Δ*phk-1*	NCU05151	Δ*phk-1::hph mat A*	FGSC 13482 (Colot et al., 2006)
ΔNCU03803	NCU03803	ΔNCU03803*::hph mat A*	FGSC 18649 (Colot et al., 2006)
ΔNCU03803	NCU03803	ΔNCU03803*::hph mat a*	FGSC 18648 (Colot et al., 2006)
ΔNCU05576	NCU05576	ΔNCU05576*::hph mat A*	FGSC 14703 (Colot et al., 2006)
ΔNCU05576	NCU05576	ΔNCU05576*::hph mat a*	FGSC 14702 (Colot et al., 2006)
Δ*sodh-2*	NCU07022	Δ*sodh-2::hph mat A*	FGSC 20151 (Colot et al., 2006)
Δ*ccl-1*	NCU02734	Δ*ccl-1::hph mat A*	FGSC 11873 (Colot et al., 2006)

Reference:

Colot, H. V., et al., 2006. A high-throughput gene knockout procedure for Neurospora reveals functions for multiple transcription factors. Proceedings of the National Academy of Sciences. 103, 10352-10357.

McCluskey, K., et al., 2010. The Fungal Genetics Stock Center: a repository for 50 years of fungal genetics research. Journal of biosciences. 35, 119-126.
